# The History of Lentil (*Lens culinaris* subsp. *culinaris*) Domestication and Spread as Revealed by Genotyping-by-Sequencing of Wild and Landrace Accessions

**DOI:** 10.3389/fpls.2021.628439

**Published:** 2021-03-25

**Authors:** Marta Liber, Isabel Duarte, Ana Teresa Maia, Hugo R. Oliveira

**Affiliations:** ^1^Interdisciplinary Center for Archaeology and Evolution of Human Behavior (ICArEHB), Universidade do Algarve, Faro, Portugal; ^2^Department of Biomedical Sciences and Medicine (DCBM), Universidade do Algarve, Faro, Portugal; ^3^Centre for Biomedical Research (CBMR), Universidade do Algarve, Faro, Portugal; ^4^Algarve Biomedical Center (ABC), Universidade do Algarve, Faro, Portugal

**Keywords:** plant domestication, legumes, biodiversity, genomics, introgression, adaptation

## Abstract

Protein-rich legumes accompanied carbohydrate-rich cereals since the beginning of agriculture and yet their domestication history is not as well understood. Lentil (*Lens culinaris* Medik. subsp. *culinaris*) was first cultivated in Southwest Asia (SWA) 8000–10,000 years ago but archeological evidence is unclear as to how many times it may have been independently domesticated, in which SWA region(s) this may have happened, and whether wild species within the *Lens* genus have contributed to the cultivated gene pool. In this study, we combined genotyping-by-sequencing (GBS) of 190 accessions from wild (67) and domesticated (123) lentils from the Old World with archeological information to explore the evolutionary history, domestication, and diffusion of lentils to different environments. GBS led to the discovery of 87,647 single-nucleotide polymorphisms (SNPs), which allowed us to infer the phylogeny of genus *Lens*. We confirmed previous studies proposing four groups within it. The only gene flow detected was between cultivated varieties and their progenitor (*L. culinaris* subsp. *orientalis*) albeit at very low levels. Nevertheless, a few putative hybrids or naturalized cultivars were identified. Within cultivated lentil, we found three geographic groups. Phylogenetics, population structure, and archeological data coincide in a scenario of protracted domestication of lentils, with two domesticated gene pools emerging in SWA. Admixed varieties are found throughout their range, suggesting a relaxed selection process. A small number of alleles involved in domestication and adaptation to climatic variables were identified. Both novel mutation and selection on standing variation are presumed to have played a role in adaptation of lentils to different environments. The results presented have implications for understanding the process of plant domestication (past), the distribution of genetic diversity in germplasm collections (present), and targeting genes in breeding programs (future).

## Introduction

Cultivated lentil (*Lens culinaris* subsp. *culinaris*) is a diploid (2*n* = 14), self-pollinating, and annual legume. With 6.3 million tons produced globally, in 2018, it constitutes an important source of dietary protein^1^. Lentil cultivation increases soil fertility via nitrogen fixation. Two broad varietal types of lentils are recognized based on morphological traits: the large-seeded *macrosperma* and the small-seeded *microsperma*; with a wide diversity of seed color and nutrient content (Singh et al., 2014). The taxonomy of the genus *Lens* has long been a matter of debate, with genetic, biochemical, morphological, plastid, and hybridization data providing conflicting results regarding its classification at the species and subspecies levels (Van Oss et al., 1997; Ferguson et al., 2000; Fratini and Ruiz, 2006; Suvorova, 2014; Koul et al., 2017). The more widely accepted taxonomy recognizes four species: *L. culinaris* with four subspecies (subsp. *culinaris*, subsp. *orientalis*, subsp. *odemensis*, and subsp. *tomentosus*), *Lens lamottei, Lens ervoides*, and *Lens nigricans* (Wong et al., 2015; Koul et al., 2017).

Cumulative evidence indicates that *L. c.* subsp. *orientalis* (henceforth referred as *orientalis*) is the wild progenitor of cultivated *L. c.* subsp. *culinaris* (henceforth referred to as *culinaris*). *Orientalis* presently occurs in Southwest Asia (SWA) and, occasionally, in Central Asia and Cyprus (Zohary et al., 2012). The other wild species are distributed throughout the Mediterranean Basin: (i) *L. ervoides* is found in Israel, Syria, Turkey, the Adriatic Coast, Southern Italy, and, rarely, in Spain and Algeria; (ii) *L. nigricans* is found in Southern Europe from Spain to Turkey, the Crimean Peninsula, Georgia, and, occasionally, in Morocco and Algeria; (iii) *L. lamottei* is found predominantly in Morocco (Davies et al., 2007; Coyne and McGee, 2013). All crop wild relatives (CWR) co-occur in southeastern Turkey, Syria, Israel, Palestine, and Jordan (i.e., Levant); it is also there that the earliest archaeological evidence for lentil domestication can be found (Coyne and McGee, 2013). The possibility that hybridization barriers might not have been strong enough to prevent different *Lens* CWR to contribute to the domesticated gene pool has not been thoroughly investigated.

Lentil was one of the first plants domesticated by humans, in SWA, together with wheat and barley, although the precise location(s) where this could have happened is still uncertain. Wild lentils were gathered, by humans in the region, as early as the Upper Paleolithic, as attested by the Ohalo II (Israel, 23,000 BP), Abu Hureyra (Syria, 13,400–11.350 BP), and Mureybit (Syria, 11,800–11,300 BP) sites. Outside SWA, *L. nigricans* was probably gathered in Franchthi Cave (Greece, 15,500–8750 cal BP) and Grotta dell’Uzzo (Sicily, 7650–6450 cal BP; Zohary et al., 2012). There is evidence for pre-domestication cultivation of *orientalis* during the Pre-Pottery Neolithic A (PPNA; 11,600–10,800 cal BP), in the sites of Jerf el Ahmar (Syria, 11,000 cal BP) and Netiv HaGdud (Jordan Valley, 11,000 cal BP; Weiss et al., 2006). In the Pre-Pottery Neolithic B (PPNB; 10,800-8,500 BP) sites in the southern Levant, lentil is the most widespread legume (Caracuta et al., 2017). Seeds there were similar in size and shape to wild *orientalis*, but they were found in association with domesticated wheat and barley (Zohary et al., 2012). Seed size is a trait that was slow to change but is traditionally used to indicate a domesticated status. At the site of Yiftah’el (Israel, 10,100–9,700 cal BP), a hoard of more than 1 million carbonized lentils was recovered contaminated with weed seeds, suggesting that lentil was by then widely cultivated (Garfinkel et al., 1988). During the PPNB, there was an increase in lentil size culminating in the fully domesticated crop (Lucas and Fuller, 2014).

A study matching archaeobotanical findings with molecular data to determine how many times lentil may have been domesticated and pinpointing the place(s) where this domestication may have taken place is lacking, unlike for other major crops domesticated in the same area (Heun et al., 1997; Pankin et al., 2018; Trněný et al., 2018; Oliveira et al., 2020). Likewise, the genetic basis of domestication syndrome traits in lentil has not been explored. This is a topic that interests evolutionary biologists and archeologists alike. Traits include an increase in seed size, indehiscent pods, decrease in carotenoid content, loss of seed dormancy, and synchronous germination (Kluyver et al., 2013; Fernández-Marín et al., 2014). Genes controlling these traits have been already identified in legumes such as soybean, chickpea, and pea (Dong et al., 2014; Hradilova et al., 2017; Varshney et al., 2019).

When agriculture spread outside the Fertile Crescent, lentils were part of the first set of crops introduced in Europe and Egypt (Sonnante et al., 2009). In the Indian subcontinent, it was a staple for the Harappan civilization (Fuller and Harvey, 2006). By the 5th millennium BCE, lentil was already adapted to the colder and more humid environments of Central Europe, being cultivated by farmers of the Neolithic Linear Pottery Culture (LBK; Zohary et al., 2012). It is unknown if the adaptation of lentils to different environments was due to standing genetic variation in wild populations, the emergence of novel alleles, to epigenetic factors, or a combination of these. The routes by which lentils spread are assumed to have mimicked the appearance of the Neolithic package in different regions, but this is yet to be confirmed.

Next-generation sequencing coupled with complexity reduction methods such as genotyping-by-sequencing (GBS) allows for the detection of thousands of genome-wide genetic markers. This has been used to study crop domestication and evolution in rice (Kim et al., 2016), common bean (Rau et al., 2019), watermelon (Nimmakayala et al., 2014), and emmer wheat (Oliveira et al., 2020). GBS has also been used to elucidate the phylogeny of the *Lens* genus (Wong et al., 2015) and the population structure of Mediterranean lentil varieties (Pavan et al., 2019). The latter study presented inferences on the history of lentil cultivation in the Mediterranean Basin. The routes of spread of a crop can also be reconstructed from the population structure of landraces (heirloom varieties) from different regions (Oliveira et al., 2014; Lister et al., 2018). We define “*landrace*” as an accession maintained and cataloged as such by a germplasm bank, with a known historical and geographical origin, adapted to its place of provenance, associated with traditional farming systems, and lacking formal genetic improvement (Villa et al., 2005). Accessions obtained by formal crop improvement using plant breeding methods are considered as “*breeds*.”

Here, we present GBS results for cultivated lentil varieties, accessions of its known progenitor, and other wild *Lens* relatives to investigate the domestication and spread of this crop. We aimed to determine how many times *L. culinaris* may have been domesticated, in which region(s) this domestication may have happened, whether introgression from other wild *Lens* species may have occurred, and which genes may have been affected by domestication.

## Materials and Methods

### Plant Material

A set of 190 wild and cultivated accessions within the *Lens* genus was analyzed (Supplementary Table 1). The wild accessions included *L. ervoides* (11), *L. nigricans* (6), *L. odemensis* (4), *L. culinaris* subsp. *tomentosus* (2), and *L. culinaris* subsp. *orientalis* (44). These taxa are found in the Near East and thus could have been involved in lentil domestication. The cultivated *culinaris* included breeds (13), landraces (106) from Eurasia and Africa, and accessions with unknown improvement status (4). Seeds were ordered from the ICARDA (Lebanon), USDA-GRIN (United States), IPK (Germany), and Nordgen (Sweden) germplasm banks. For cultivated accessions, seeds were germinated in petri dishes and DNA was extracted from hypocotyls. For wild accessions, germination was not always successful and the number of seeds provided was small, so DNA was extracted directly from single seeds pulverized inside a tin foil wrap to prevent dust contamination. All extractions were made with the DNeasy Plant Mini Kit (Qiagen) and DNA was quantified with a Qubit dsDNA HS assay on a Qubit 2.0 Fluorometer. DNA integrity for GBS was checked by running all extractions on a 1% agarose gel. Two 96-well plates were prepared, with each well containing 300 ng of DNA.

### Genotyping-by-Sequencing

#### Library Preparation and Sequencing

Genotyping-by-sequencing was outsourced to LGC Genomics GmbH (Berlin, Germany). Libraries, including indexing, were prepared using a combination of *Pst*I and *Ape*KI restriction enzymes. Libraries were pooled and sequenced in an Illumina NextSeq 550 System (150-bp single reads). The raw sequencing reads were deposited in the ArrayExpress database at EMBL-EBI^2^ under accession number E-MTAB-9222.

#### Read Pre-processing

Demultiplexing barcoded samples and verification of restriction sites were done using the Illumina bcl2fastq2 v.2.17.1.14 software (no mismatches or Ns were allowed for inline barcodes, but Ns were allowed for restriction sites). Sequencing adapter remnants were clipped from all reads and reads with final length <20 bp or 5′ends not matching the restriction enzyme site were discarded. Further trimming, which was done with CUTADAPT v.3.2 (Martin, 2011), included removal of reads containing Ns, trimming of reads at 3′-end to get a minimum average Phred quality score of 20 (over a window of ten bases) and discarding reads with final length <20 bases.

#### GBS Clustering and SNP Calling

Combined reads were clustered with CD-HIT-EST, allowing up to 5% difference. Quality trimmed reads were aligned against a cluster reference with Bowtie2 v.2.2.3. Variant discovery and genotyping of samples were done with freebayes v.1.0.2-16 using the following parameters (–min-base-quality 10 –min-supporting-allele-qsum 10 –read-mismatch-limit 3 –min-coverage 5 –no-indels –min-alternate-count 4 –exclude-unobserved-genotypes –genotype-qualities –ploidy 2 –no-mnps –no-complex –mismatch-base-quality-threshold 10), and .vcf files were generated (henceforth referred to as Dataset 1). Different filters were subsequently applied to the VCF files using TASSEL 5 v. 5.2.52 (Glaubitz et al., 2014) and VCFtools v0.1.16 (Danecek et al., 2011). As an alternative strategy, reads were also mapped against the chickpea reference genome^3^ using BWA-MEM v.0.7.12 (Li and Durbin, 2010).

### Phylogenetic Inference

For phylogenetic analyses two inference methods were adopted for confidence and consistency: Maximum Likelihood (ML), using RAxML v.8.0.0 (Stamatakis, 2014), and Bayesian Inference (BI) python script vcf2phylip.py v.2.0, using MrBayes v. 3.2.7a (Ronquist et al., 2012). Analyses were conducted at the CIPRES Science Gateway V.3.3^4^. First, VCF files were converted to phylip and nexus formats using the python script vcf2phylip.py (Ortiz, 2019).

We determined the best-fit evolutionary model for the dataset using jModelTest v.2.1.4 (Darriba et al., 2012). For all 190 accessions, we conducted phylogenetic analyses using the general time-reversible model of nucleotide substitution including gamma-distributed rates across sites (GTR + Γ). For the *L. culinaris* subset of 157 accessions, phylogenetic analyses were carried out also accounting for the proportion of invariable sites (GTR + I + Γ model).

The best ML tree was obtained by coupling 100 rapid bootstrap iterations and searching for the best-scoring ML tree in a single RAxML run. Two independent runs, with four chains and 1 million generations each, were computed using MrBayes. Markov chains were sampled every 500 generations with swaps of states between chains being tried on each generation of the run. The burnin was set to 25%.

For the complete set, *L. nigricans* was used as the outgroup; for the *L. culinaris* set, *orientalis* accessions were used as the outgroup (shown by non-parametric means to have no allelic input from the other CWR).

### Population Structure

We used principal component analysis (PCA) and discriminant analysis of principal components (DAPC) to explore similarities between the accessions in the dataset. Firstly, the raw VCF files were read as *vcfR* objects using the vcfR package (Knaus and Grünwald, 2017) and then converted with the R package adegenet v.2.0.1 (Jombart, 2008; Jombart et al., 2010) to a *genelight* object for downstream analysis. Next, a PCA was performed in R using the *prcom*p function of the stats v.3.6.2 R package (R Core Team, 2012), with scaling and centering parameters set to TRUE. To investigate the effects of filtering in the inference of population structure, new PCAs were performed for datasets obtained with different filtering conditions for minor allele frequency (MAF), coverage, and missing data. Results were visualized in R using the following packages: ggplot2 (Wickham, 2016), plotly (Sievert et al., 2017), gridExtra (Auguie and Antonov, 2017), and RColorBrewer (Neuwirth, 2011). Additionally, non-parametric analysis of population structure with DAPC was conducted with the find.clusters function from the adegenet R package (Jombart, 2008; Jombart et al., 2010), after computing the number of principal components to be retained. All these analyses were carried in a Linux environment (Ubuntu 18.04.4 LTS), using R programming language v.3.6.3 in the integrated development environment (IDE) RStudio v.1.1.463.

As for parametric analyses, population structure was also examined using the Bayesian model-based clustering algorithm STRUCTURE 2.3.4 (Pritchard et al., 2000), with *K*-values between 1 and 20, 40,000 burnin, 80,000 MCMC iterations, and 10 independent runs for each value of *K*. The most likely values of *K* were chosen based on the Δ*K* method (Evanno et al., 2005), computed in STRUCTURE HARVESTER (Earl and Vonholdt, 2012). STRUCTURE was run for two sets of accessions: (1) the complete dataset, with 190 accessions (including all wild taxa), and (2) the *L. culinaris* accessions only, with 157 accessions (including the wild *orientalis* and the cultivated *culinaris*). The former aimed to investigate relationships across genus *Lens*, and the latter pertained to the domestication process. Q-matrixes were plotted in MS Excel and displayed on geographic maps using ArcMap v.10 (ESRI, 2011) and the R package rgdal (Bivand et al., 2015), with gstat for spatial interpolation (Gräler et al., 2016). Accessions found to be misclassified in all population structure methods were re-classified (Supplementary Table 1). Nucleotide diversity (π) (Nei, 1987), Watterson’s theta (θ) (Watterson, 1975), and Tajima’s D (Tajima, 1989) were computed for each species and cluster detected by different methods using TASSEL 5. Pairwise *F*_*ST*_ between taxa was calculated with packages adegenet and vcfR (Jombart, 2008; Knaus and Grünwald, 2017).

### Inferring Domestication History

The four-taxa ABBA-BABA test was used to detect introgression between wild *orientalis* and the genetic clusters of domesticated *culinaris* defined by population structure methods (Li et al., 2014; Owens et al., 2016). Dsuite v.0.1 was employed on *dataset 1* to calculate the *D*-statistic from the single-nucleotide polymorphism (SNP) data (Malinsky, 2019). *L. ervoides* was here used as the outgroup.

To test isolation-by-distance in the dispersal of domesticated lentils outside the Fertile Crescent in SWA, the correlation between pairwise genetic distances and pairwise geographic distances between individual accessions was performed with a Mantel test, and a density plot was obtained with the MASS package v. 7.3-53.1 (Ripley et al., 2013). This was done for all accessions and for accessions within the clusters defined by population structure methods (*see above*).

### Genetic Basis of Lentil Domestication and Adaptation

We used GWAS to identify markers associated with the domesticated status (*domestication* as a trait with two states: wild vs. domesticated). Potential hybrid accessions and outliers in population structure analysis were excluded (see Supplementary Table 1). *Dataset 1* was further filtered in TASSEL 5 to keep only *L. culinaris* accessions (subsp. *culinaris* and *orientalis*), and missing data were also removed (9617 SNPs). A mixed linear model (MLM), using the first five components of a PCA as input for population structure and a kinship matrix (Q + K model), was also computed in TASSEL 5. A Bonferroni correction with α = 0.005 was used to highlight significant marker–trait associations.

We also investigated the genetic basis of lentil adaptation to different environments. Here, we filtered *Dataset 1* considering only cultivated *culinaris* landraces and without missing data (12,924 SNPs). Four environmental variables were tested as proxies for temperature and water stress: (1) “maximum temperature of the warmest month,” (2) “minimum temperature of the coldest month,” (3) “precipitation of the wettest quarter,” and (4) “precipitation of the driest quarter.” The values of temperature and precipitation for each location were extracted from the bioclimatic variables available at WorldClim^5^ with a 5-min spatial resolution and using the raster and rgdal packages in R (Hijmans et al., 2016). In TASSEL 5, GWAS was carried out to perform an association scan for each of the four climatic variables, using a generalized linear model (GLM) (Olatoye et al., 2018).

Single-nucleotide polymorphisms noted down by these three methods had their flanking sequences retrieved from the BAM files. To infer the putative role of these SNPs, their sequences were submitted to a BLAST search using the Standard Nucleotide BLAST tool at the NCBI platform^6^.

## Results

### SNP Detection

The use of *Pst*I and *Ape*KI restriction enzymes was found to be suitable for complexity reduction in a set of 190 wild and cultivated lentils. A total of 384 million reads were obtained with an average of 1.7 million quality trimmed reads per sample. There was a slightly higher average number of reads for domesticated lentil than for wild lentil accessions (Table 1), but the method was effective in detecting variation for the different *Lens* species. The number of reads varied from 6 million in accession PI 297779 (*culinaris*) to 0.3 million in PI 572329 (*L. ervoides*) (Supplementary Table 1). While mapping of reads against a cluster reference with Bowtie2 v.2.2.3 yielded a mapping rate of 98.89% (87,647 SNPs across all samples with the filtering conditions of *Dataset 1*), mapping of reads against the chickpea reference genome was met with a mapping rate of 44.10% (1423 SNPs across all samples). We therefore used only the cluster reference method.

**TABLE 1:** Genotyping-by-sequencing (GBS) statistics and genetic diversity measures based on 87,647 SNPs for species (bold) and selected groups within the *Lens* genus.

**TAXA**	**Number of Taxa**	**Quality trimmed Reads**	**Proportion of missing data**	**Average MAF**	**PiPerBP**	**ThetaPerBP**	**Tajima*D***
***Lens culinaris* subsp. *culinaris***	123	**1828839**	**0.482**	**0.125**	**0.107**	**0.136**	**−0.786**
Group A (light blue)	40	1644022	0.493	0.089	0.095	0.118	**−**0.848
Group B (red)	31	2033899	0.497	0.086	0.103	0.116	**−**0.485
Group C (purple)	42	1865280	0.487	0.087	0.078	0.114	**−**1.338
***Lens orientalis* subsp. *orientalis***	**34**	**1511361**	**0.568**	**0.108**	**0.111**	**0.172**	**−1.669**
Group D1	11	1564892	0.577	0.061	0.105	0.119	**−**0.844
Group D2	23	1350031	0.563	0.092	0.105	0.161	**−**1.882
***Lens orientalis* subsp. *odemensis***	**4**	**1141630**	**0.642**	**0.020**	**0.074**	**0.061**	**0.148**
***Lens ervoides***	**16**	**1127611**	**0.668**	**0.025**	**0.063**	**0.062**	**0.145**
***Lens nigricans***	5	1384657	0.739	0.019	0.098	0.089	**−**0.631
**All wild**	55	1291315	0.607	0.139	0.154	0.217	**−**1.303

### Exploratory Analysis

We explored population structure by looking at clusters revealed by the PCA, using datasets with different filtering conditions for minimum number of reads for each SNP (between 5× and 8×), proportion of missing data, number of accessions where a SNP is detected, and MAF values (Supplementary Figure 1). Regardless of the number and quality of SNPs used, two main groups were detected: one included *orientalis* together with *culinaris*, and the other comprised *L. c.* subsp. *odemensis, L. ervoides*, and *L. nigricans*; each of those groups comprise discrete sub-groups of their own. However, the general dispositions of the sub-groups and their proximity to *culinaris/orientalis* changed significantly depending on the different SNP filtering options. For example, in filtering conditions A and C in Supplementary Figure 1, *L. nigricans* was the more distinct (distant) species from the *culinaris/orientalis* complex, whereas in conditions I and K, that position was taken by *L. ervoides*. This indicates that filtering conditions applied on GBS-developed SNPs can indeed affect data interpretation (Schilling et al., 2014). We therefore chose to conduct downstream analysis on two datasets (C and K in Supplementary Figure 1, respectively); the first was the cleaned raw data (*Dataset 1*) and the second had more conservative filtering criteria (keeping only SNPs with a MAF of 0.05 and observed in 2/3 of the accessions, which resulted in a dataset with 8791 SNPs).

**FIGURE 1:**
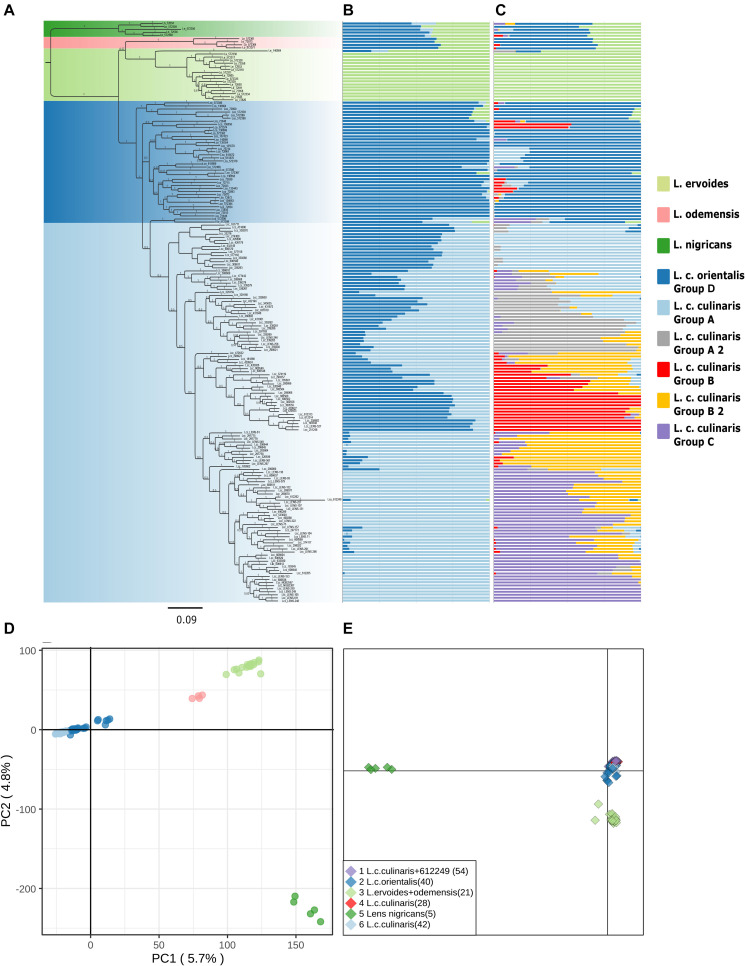
Phylogeny and population structure analysis of 190 accessions of wild and cultivated lentil based on 87,647 SNPs revealed by GBS. **(A)** MrBayes generated phylogeny. **(B)** STRUCTURE *K* = 3 model. **(C)** STRUCTURE *K* = 7 model. **(D)** First and second principal components of a PCA. **(E)** DAPC analysis.

The accessions clustering allowed us to identify misclassified accessions, and what could be inter-specific hybrids (Supplementary Table 2). Supplementary Figures 2, 3 show the PCA and STRUCTURE results with the complete set of accessions for the original seed bank classifications. For example, accession IG72553 was classified as *L. nigricans*, but in the PCA, it is clearly grouped with the bulk of *L. ervoides* accessions (top right panel in Supplementary Figure 2). In the STRUCTURE *K* = 3 and *K* = 7 models with the complete set of accessions, IG72553 belongs to the light green cluster, like all other *L. ervoides* accessions (Supplementary Figure 3). Likewise, phylogenetic analysis places this particular accession in the same clade as *L. ervoides* (Figure 1). Similarly, accession PI612249, classified as *orientalis* from Turkey, shows up in STRUCTURE and in the phylogeny as a misclassified *culinaris*, but in the PCA, it shows equally distant to both *orientalis* and *culinaris* (top left panel in Supplementary Figure 2). If this accession is in fact a hybrid, a naturalized cultivar, or a misclassified accession, remains to be determined and, therefore, it was excluded from subsequent analyses of *L. culinaris* accessions. Based on these criteria, nine accessions were re-classified, and four were removed from downstream STRUCTURE analysis (Supplementary Table 2).

### Phylogenetic Inference

Exploratory analysis using Maximum Likelihood (ML) methods (RAxML) for *Dataset 1* with the complete accession panel indicated the general time-reversible, with rate variation across sites (GTR + Γ), as the most likely substitution model; we rooted the topology with *L. nigricans* as the outgroup (Supplementary Figure 4). The Bayesian phylogeny inferred (with MrBayes) presented a somewhat similar topology (Figure 1A). Both *orientalis* (in dark blue) and *culinaris* (in light blue) were placed in a separate clade from wild *L. nigricans* (in dark green), *L. ervoides* (in light green), and, notably, *L. c.* subsp. *odemensis* (in pink). *Subspecies odemensis* (in pink) was inferred to be sister to *ervoides* (in light green) in the Bayesian topology (Figure 1A), while in the ML topology (Supplementary Figure 4), it was placed as sister to the *L. culinaris* clade (dark and light blue). Within the *L. culinaris* clade, all *culinaris* accessions are clustered together within *orientalis* (except for putative hybrids, as revealed by the STRUCTURE software).

**FIGURE 2:**
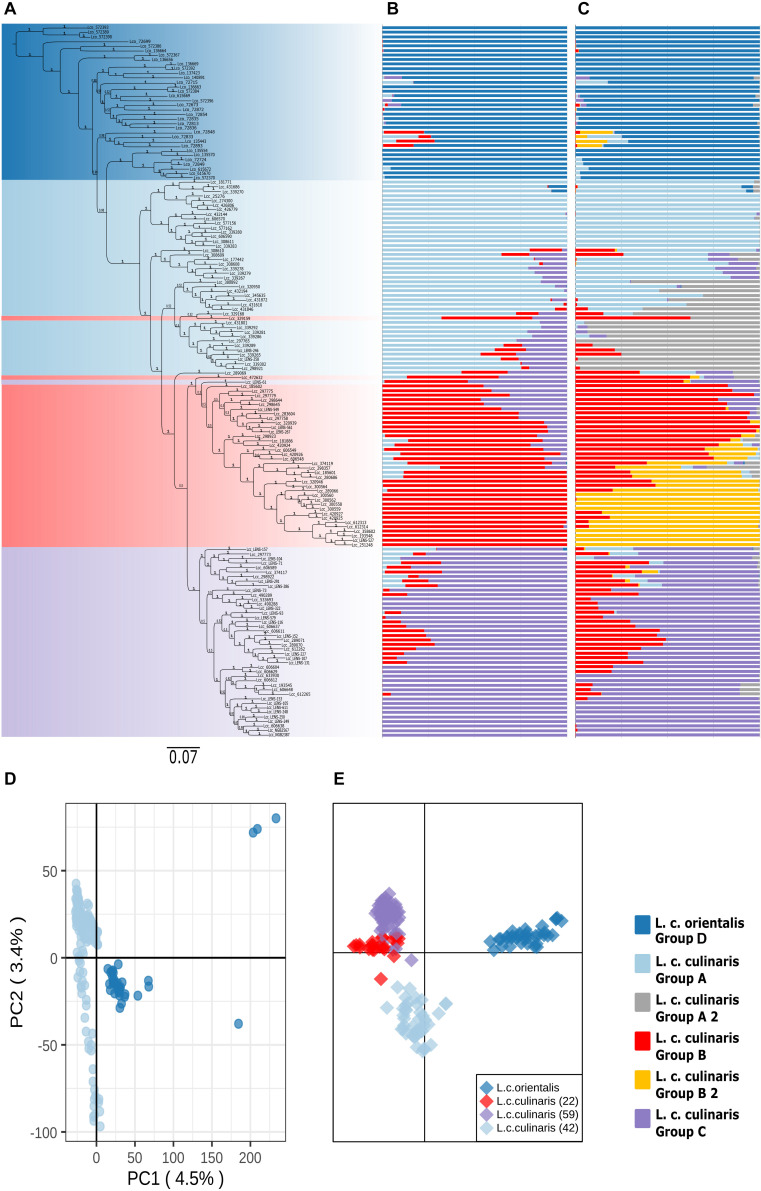
Phylogeny and population structure analysis of 157 accessions of *L. culinaris* (wild *orientalis* and cultivated *culinaris*) based on 87,647 SNPs revealed by GBS. **(A)** MrBayes generated phylogeny. **(B)** STRUCTURE *K* = 4 model. **(C)** STRUCTURE *K* = 6 model. **(D)** First and second principal components of a PCA. **(E)** DAPC analysis.

When a phylogeny with only *L. culinaris* (subsp. *orientalis* and *culinaris*) accessions was computed, domesticated *culinaris* accessions were in a clade nested within wild *orientalis* ones (Figure 2A). In this Bayesian phylogeny, seven *orientalis* accessions composed a sister clade to the domesticated ones, and these included accessions from Central Asia, as well as an accession from Cyprus and another from Turkey; this Central Asian clade was not retrieved in the ML analyses. With regard to the *culinaris* accessions, the three major groups detected by parametric and non-parametric means corresponded, roughly, to a three-clade grade and to two terminal clades.

### Population Structure

Parametric and non-parametric analysis revealed broadly the same population structure (Figures 1, 2). In the PCA, four groups were identified: *L. culinaris* (both subsp. *orientalis* and *culinaris*), *L. nigricans*, *L. ervoides*, and *L. c.* subsp. *odemensis* (Figure 1C). The latter subspecies was distinctly separated from the former accessions in the PCA, especially with regard to the third principal component (Supplementary Figure 5) and could be classified as a separate species. In the DAPC, however, *L. ervoides* and *L. c. odemensis* were clustered closely together (Figure 1E). In these groups, the highest diversity was found within *orientalis* for both measures of nucleotide diversity (π = 0.111) and nucleotide polymorphism (θ = 0.172). The smallest nucleotide diversity was observed within *L. ervoides* (π = 0.063), although nucleotide polymorphism levels were similar to those of *L. c.* subsp. *odemensis* (θ = 0.062 and θ = 0.061, respectively). The same result was obtained when the original *Dataset 1* was filtered to exclude missing data (4421 SNPs), meaning missing data do not seem to be misleading the comparisons of genetic diversity between taxa (Supplementary Table 3). Excluding missing data did, however, affect Tajima’s *D* calculation. When this index was computed in *Dataset 1* (87,647 SNPs), *L. c.* subsp. *odemensis* and *L. ervoides* had positive values and all the remaining taxa were in the negative (Table 1). When the *Dataset 1* without missing data was used, all taxa had negative values (Supplementary Table 3). The largest pairwise *F*_*ST*_ was between *L. ervoides* and *L. nigricans*, and the smallest one was between *culinaris* and *orientalis*; the same was true for Nei’s genetic distance (Table 2). In the STRUCTURE analysis with the complete set, *K* = 3 and *K* = 7 were the preferred models, according to Evanno’s Δ*K* method. Clusters identified in these models correspond to the grades and clades seen in the phylogenetic analyses. When *K* = 3, *L. ervoides* belonged to its own cluster (light green), so did *orientalis* (dark blue), whereas *L. nigricans* and *L. c.* subsp. *odemensis* got their alleles, on one hand, from the *ervoides* group and, on the other, from the *orientalis* group (Figure 1B). Also, when *K* = 3, *culinaris* accessions got a varying degree of alleles from the *orientalis* group. Accessions that in the *K* = 7 model belonged to clusters B2 (orange) and C (purple) had the lowest proportion of *orientalis* alleles (Figures 1B,C).

**TABLE 2:** *F*_*ST*_ (below diagonal) and Nei’s genetic distance (above diagonal) between species within the *Lens* genus based on 87,647 SNPs.

	***Lens culinaris* subsp. *orientalis***	***Lens culinaris* subsp. *culinaris***	***Lens ervoides***	***Lens nigricans***	***Lens culinaris* subsp. *odemensis***
*Lens culinaris* subsp. *orientalis*	–	0.0089	0.0941	0.2056	0.0839
*Lens culinaris* subsp. *culinaris*	0.1794	–	0.0986	0.2085	0.0892
*Lens ervoides*	0.6320	0.7459	–	0.2289	0.1299
*Lens nigricans*	0.7660	0.8522	0.8710	–	0.2461
*Lens culinaris* subsp. *odemensis*	0.5691	0.7179	0.7996	0.8475	–

When only *L. culinaris* (*orientalis* and *culinaris*) accessions were analyzed, a complete separation between the wild and cultivated forms was observed. In the PCA, four *orientalis* accessions (three from Turkey, one from Syria) were considered distinct from the rest along both axis: PI572389, PI572390, PI572393, and IG72699 (Figure 2D). In the STRUCTURE analysis, *K* = 4 and *K* = 6 were the most likely models (Figures 2B,C). These models considered *orientalis* as a distinct cluster (named group D), with characteristic genetic identity, i.e., only six accessions showed more than 10% of their alleles coming from *culinaris* clusters: IG72715 (Syria), IG72848 (Jordan), IG72833 (Turkey), IG135443 (Syria), IG72893 (Syria), and IG72715 (Syria) (Figure 2B). Also, notably, accession IG136658 (Israel) appeared in the model *K* = 4 as a hybrid between *orientalis* and *culinaris* (Supplementary Figure 6), and the same was applied to accession PI572374 (Iran) (Supplementary Figure 3B). Supplementary Figure 7 shows that *orientalis* accessions with shared ancestry with *culinaris* accessions are located mostly in the southern Levant.

Within *culinaris*, accessions were grouped in accordance with the STRUCTURE genetic analysis for *K* = 4. We named these group A (light blue), group B (red), and group C (purple). Groups A and B were further divided in the *K* = 6 model (Figures 2B,C). Except for three accessions, these clusters corresponded to clades in the phylogenetic tree. Many accessions got proportional membership to more than one cluster with shared alleles being more common between groups B (red) and C (purple).

**FIGURE 3:**
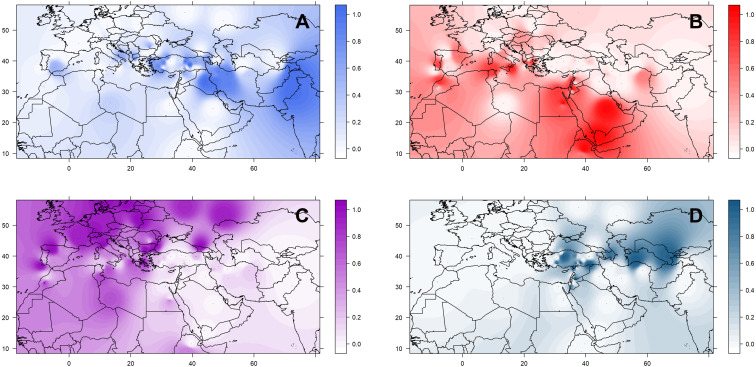
Geographical distribution of the four groups **(A–D)** of *L. culinaris* identified in the STRUCTURE model *K* = 4. Spatial interpolation is based on the *Q*-matrix of proportional memberships of individual accessions to each of the four groups.

The geographic distribution of these groups is not random (Supplementary Figure 8). Accessions belonging to the three *culinaris* groups (A, B, and C) can be found throughout the range of lentil cultivation. However, regional patterns were revealed by the interpolation of proportional membership with spatial occurrence (Figure 3). Group A was predominantly found in Turkey, expanding eastwards toward Iran/Iraq, Central Asia, and India. When subdivided into groups A1 and A2, in the STRUCTURE *K* = 6 model, group A1 (light blue) was present in southern Turkey, Syria, and Iraq, whereas group A2 (gray) extended westwards into Greece and Italy, and it was more frequent in northern Turkey and Iran (Supplementary Figure 9). Group B was distributed along the southern Levant, Arabian Peninsula, Horn of Africa, and northern Africa. When subdivided into groups B1 and B2 in the STRUCTURE *K* = 6 model, group B2 (orange) was restricted to the southern Levant, Arabian Peninsula, and Horn of Africa (Supplementary Figure 9). Group C was only found outside the Fertile Crescent and includes all Central European accessions and many of the Mediterranean Basin ones (Figure 3). Although differences in genetic diversity between the three major groups were small, both π and θ were smaller in group C than in the other groups (Table 1). Pairwise *F*_*ST*_ was lowest between group A and the *orientalis* accessions (group D), lower in fact than between any group of *culinaris* accessions (Supplementary Table 4).

To identify geographically restricted accessions of *orientalis* that could pinpoint the place of lentil domestication, we run STRUCTURE on the group D set of *orientalis* accessions. We identified two clusters: one predominantly occurring in Central Asia, Cyprus, and southeastern Turkey (group D1), and another one mainly found in Syria, Jordan, the Caucasus, and western Turkey (group D2; Supplementary Figure 10). Group D2 *orientalis* accessions had a lower Pairwise *F*_*ST*_ in relation to the *culinaris* groups A, B, and C than group D1 *orientalis* (Supplementary Table 5). The lowest inter-subspecies Pairwise *F*_*ST*_ was found between *orientalis* group D2 and *culinaris* group A, suggesting that these two populations are the most genetically similar (Holsinger and Weir, 2009).

Some of the accessions classified as breeds, improved varieties, or with unknown improvement status were indistinguishable from landraces from the same regions, for example, *Lens*-105 (Germany) or PI 345635 (Armenia) (Supplementary Figure 11). This suggests that they were developed from local landraces. Others were most likely bred from landraces originating in different regions (e.g., PI 289066, from Hungary, belonged to the red group B cluster), and some showed signs of genetically distinct parental lines (e.g., PI 289070, from Hungary, and PI 374117, from Morocco).

### Domestication History

**FIGURE 4:**
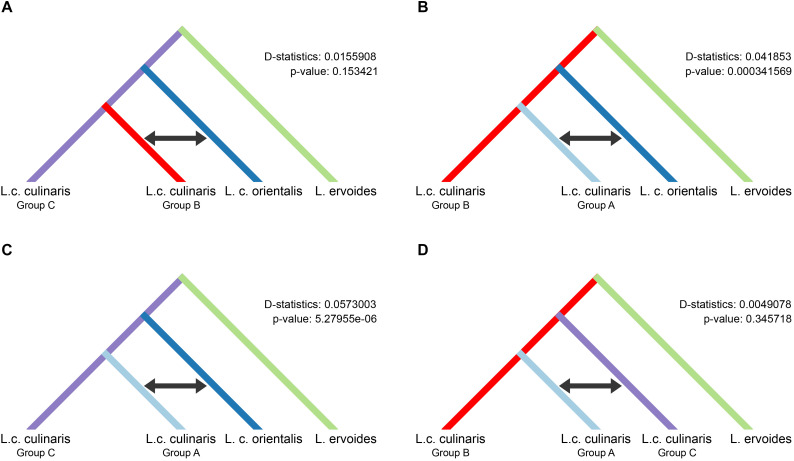
Results of the four-taxon ABBA-BABA test used to detect introgression between wild *orientalis* and groups of *culinaris* **(A–C)** and between *culinaris* groups **(D)**. Groups were defined in the STRUCTURE *K* = 4 model. The black arrow indicates the groups between which gene flow is detected.

We used the four-population (P1–P4) ABBA-BABA test, under different scenarios, to investigate the possibility of gene flow between each of the three groups of domesticated *culinaris* with respect to the wild *orientalis*. We assumed P1 and P2 to be two of the three domesticated lentil groups singled out by STRUCTURE, P3 was the wild *orientalis*, and P4 was *L. ervoides* (here as the outgroup). In a scenario of solely incomplete lineage sorting (ILS), and in the absence of gene flow between either domesticated group (P1 or P2) with wild orientalis (P3), the value of the *D*-statistic is expected to be zero. If there were to be more gene flow between P2 and P3 than between P1 and P3, *D* would be expected to be negative, and if gene flow were more intense between P1 and P3, *D* would be positive. In all the scenarios we tested, the *D*-statistic was positive, albeit very low (<0.06), indicating a small amount of gene flow between the *orientalis* and the *culinaris* groups A and B (Figure 4). The highest proportion of gene flow was observed between *culinaris* group A and *orientalis* when compared with the other groups (Figures 4B,C). A residual amount of gene flow between group B and *orientalis* was detected, but introgression between group C and *orientalis* was not identified. Presently, it is not possible to infer if the gene flow resulted from cultivated lentil into wild stands or vice-versa. Within cultivated lentil, gene flow between the different groups did occur, although at residual levels (*D*-statistic = 0.005), and more between groups A and C, than between groups B and C (Figure 4D). When *L. nigricans* is used as the outgroup the picture remains unchanged, except that, although still residual (*D*-statistic = 0.004*; p-*value = 0.362), more introgression is detected between groups B and C than between groups A and C (Supplementary Figure 12).

**FIGURE 5:**
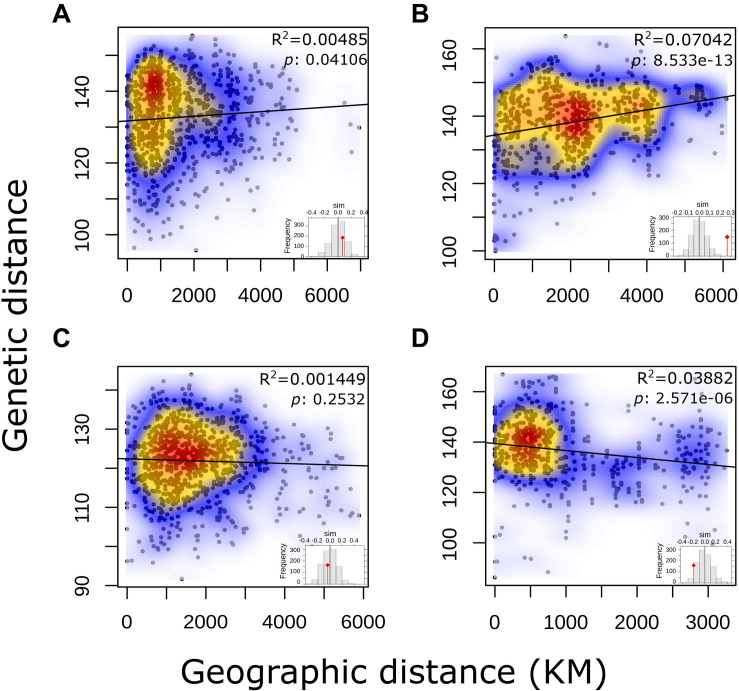
Output of a Mantel test to determine Isolation-by-Distance in each of the STRUCTURE *K* = 4 defined groups of *L. culinaris* accessions.

No correlation for pairwise genetic and geographic distances was observed for either the complete panel of *L. culinaris* accessions or for the groups identified by population structure methods (Figure 5). This indicates that geographic distance between accessions is not correlated with genetic distances. When accessions without precise provenance indicated in the passport data (column “Notes” on Supplementary Table 1) were removed, *R*^2^ remained low.

### GWAS

An MLM Genome-Wide Association Study (GWAS) computed to identify markers associated with the domesticated traits yielded only three SNPs above the Bonferroni correction threshold (Supplementary Table 6). A BLAST search of the SNPs flanking sequences produced only one hit for a putative protein in chickpea, so the function of these markers could not be inferred.

A GWAS using a GLM was computed in order to detect SNPs associated with adaptation to four climatic variables. No markers were found associated with “precipitation of the driest quarter,” nor for “minimum temperature of the coldest month.” Three markers were identified for “precipitation of the wettest quarter” and in two of them, the alternate allele was observed only in lentil accessions of group B, which is distributed in very hot or desert areas (Figure 3B). Homologous sequences were found in several legume species coding for an amino acid permease and an ABC transporter (Supplementary Table 6). For adaptation alleles, we looked at which allele (reference, alternate, or both) was present in the set of *orientalis* accessions. This was meant as a means to infer if adaptation was mostly based on standing variation, existing in the wild progenitor, or whether novel mutations had had to evolve in the domesticated crop. In the case of two of these three markers, only the reference allele was present in the *orientalis* accessions, and in the other two, both alleles could be found in the wild. For the “maximum temperature of the warmest month” trait, two SNPs in the same contig, with the alternate allele present in the same accession, were detected. These accessions were from Egypt and Saudi Arabia, and this mutation was not present in any of the *orientalis* accessions. Thus, this contig could be a good candidate for an adaptative mutation emerging after domestication. This sequence codes for a zinc finger BED domain-containing protein DAYSLEEPER in both chickpea and soya.

## Discussion

### Methodological Issues

Our work confirms the usefulness of GBS to investigate crop domestication histories as well as taxonomic relationships at the genus level and below. GBS was able to detect SNPs for the particular set of individuals used, thus avoiding the ascertainment bias that occurs when panels of SNPs developed in a small number of commercial varieties are used to screen landraces or wild plants (Heslot et al., 2013; Chu et al., 2020). Other than in lentil (Wong et al., 2015), GBS has been used to investigate phylogenetic relationships within carrot (Arbizu et al., 2016), amaranth (Stetter and Schmid, 2017), coffee (Hamon et al., 2017), and wheat (Hyun et al., 2020).

We attempted to map reads against a well-annotated reference genome. During the time this work was developed, there was no assembled lentil genome, and we therefore chose the chickpea genome, because it has a similar domestication history to that of lentil (Zohary et al., 2012). However, the use of a BWA pipeline to map reads against the chickpea genome produced 61× less SNPs than a pipeline without a reference genome. This is in contrast with previous observations where pipelines that use a reference genome identified more SNPs than two alternative pipelines that do not require a reference genome (Torkamaneh et al., 2016). This discrepancy is probably due to the lack of synteny between the lentil and the chickpea genomes, as they belong to different tribes within the Fabaceae family (Fabeae and Cicereae, respectively) (Smıkal et al., 2015).

Using a cluster reference method, instead of a reference genome, we were still able to generate sound phylogenetic inferences from GBS data (Figure 1). The use of different pipelines and filtering criteria on GBS data is known to affect the number and quality of SNPs identified (Torkamaneh et al., 2016; Yu et al., 2017; Loureiro et al., 2020b). Our filtering conditions produced widely different numbers of SNPs requiring decisions to be made between loose or more restrictive filtering conditions (more coverage, less missing data, higher MAFs) for different types of analyses. Nonetheless, it has been observed that filtering conditions do not, in general, affect determination of population structure or phylogenetic inference (Loureiro et al., 2020a; Oliveira et al., 2020). The clustering of accessions based on PCAs obtained from datasets with different numbers of SNPs was very similar (Supplementary Figure 1), with the four basic groups in the *Lens* genus distinctively recovered time and again. For questions pertaining to population structure and phylogenetic relationships, filtering conditions did not affect the results. Population structure analyses and phylogenies inferred were similar to previous studies: four or five groups, when different species within *Lens* are considered (depending on whether *orientalis* and *culinaris* are split or not) (Alo et al., 2011; Ogutcen et al., 2018; Dissanayake et al., 2020); and three major clusters within cultivated lentils (Khazaei et al., 2016; Koul et al., 2017).

The very clear separation of accessions by species in phylogenetic and population structure analyses allowed the identification of misclassified accessions. We found nine accessions, all wild, where the species reported in the passport data did not match their placement in PCA, STRUCTURE, or phylogenetic trees. Misclassification of samples is a recurrent issue with germplasm accessions (Mason et al., 2015). In lentil studies, Dissanayake et al. (2020) observed 22 misclassified accessions out of 467 lentil accessions, and 4 out of 83 accessions were identified as erroneously classified by Wong et al. (2015). Less straightforward is how to deal with accessions that are putative hybrids between different groups or that could have been affected by gene flow along their evolutionary past. These could be informative about introgression processes, or mere methodological noise. We opted to flag them but exclude them from the downstream analyses of *L. culinaris* accessions (Supplementary Table 2).

### The *Lens* Genus

Our results corroborate previous studies that identified four major groups within the *Lens* genus (Wong et al., 2015; Dissanayake et al., 2020). Interestingly, the STRUCTURE software did not so clearly single out these groups (Figures 1B,C). Here, *L. c.* subsp. *odemensis* and *L. nigricans* seemingly appear to be *L. ervoides* and wild *L. culinaris* hybrids. This is unlikely to reflect a biological reality. It has been shown that for this type of Bayesian approach, groups with a small number of samples or with small genetic drift are likely to present as admixed between other groups in the panel (Lawson et al., 2018). This was likely the case for these two taxa in our panel. *Orientalis* and *culinaris* were placed in the same clade in the phylogeny and clustered together in the PCA and DAPC (Figure 1), separately from all other taxa. They were, however, distinct in the *K* = 7 STRUCTURE runs, with the whole accession panel (Figure 1C), and in the runs including only *L. culinaris* (Figure 2).

Alo et al. (2011) and Wong et al. (2015) considered *L. nigricans* to be the most distantly related species to the domesticated *culinaris*. Since we did not sample outside genus *Lens*, we could not determine which species was furthest to domesticated culinaris and, thus, chose to follow the existing consensus (see Supplementary Figure 13 for alternative rootings). Based on our phylogenetic and population genetics analyses, both *L. nigricans* and *L. ervoides* are quite distinct from the domesticated gene pool. *L. ervoides* is inferred as sister to *L. c.* subsp. *odemensis* (Figure 1A), resembling the topology obtained by Dissanayake et al. (2020). Computation of Nei’s genetic distances and *F*_*ST*_ values indicate *L. nigricans* as the most distinct taxon from *L. culinaris* (Table 2). Wong et al. (2015) considered *tomentosus* as part of the same gene pool as *culinaris* and *orientalis*. We only had two accessions of *tomentosus* in our panel and both were clustered with *L. ervoides* accessions (Supplementary Figure 2). Most likely, these two accessions were mislabeled, and this observation does not necessarily support a case for *tomentosus* being considered part of the *L. ervoides* gene pool.

The negative values of Tajima’s *D* suggest an abundance of rare alleles that seem to indicate recovery from a recent population bottleneck or a population expansion (Jensen et al., 2005). In the case of crops, this effect has been observed for GBS-generated SNPs and interpreted as a bottleneck following domestication from a population that had a narrow genetic pool during domestication (Nimmakayala et al., 2016). This conclusion must be met with caution as demographic events and selection can be difficult to distinguish in the absence of genome-wide data (Cortés and Blair, 2018). In any case, in our data, negative Tajima’s *D* values were observed in all taxa, not just domesticated types. Sequencing of domestication-related genes in cultivated and wild *Lens* could elucidate this aspect. It is noteworthy that filtering missing data out for *Dataset 1* changed the signal of Tajima’s *D* values for *L. c.* subsp. *odemensis* and *L. ervoides*. The effects of SNP filtering in GBS data need to be considered when computing this statistic from GBS data.

### Domestication and Spread of Lentil Cultivation

Our data indicate that *orientalis* is the wild progenitor of the cultivated lentil, with other wild *Lens* species having residual or no input into the cultivated gene pool. The analysis of population genetic structure *K* = 3 model indicates a wide sharing of alleles between *orientalis* and *culinaris* accessions from SWA and the Mediterranean Basin.

When assigning geographic information to the genetic data, our results suggest a single origin for lentil domestication but fail to pinpoint a precisely localized origin for said domestication. In the phylogenetic analyses, all *culinaris* are clustered together and nested within *orientalis*. In accord with the view that monophyly indicates a single domestication event, as opposed to multiple ones, our data support a single origin for lentil, similar to the proposed origins of einkorn, emmer wheat, barley, maize, pearl millet, soja, carrot, and sunflower (Luo et al., 2007; Ross-Ibarra and Gaut, 2008; Honne and Heun, 2009; Guo et al., 2010; Dussert et al., 2015; Arbizu et al., 2016; Park and Burke, 2020). Notwithstanding, this interpretation has been disputed on methodological and biological grounds for other SWA crops (Allaby et al., 2008; Brown et al., 2009; Fuller et al., 2011). In the case of lentil, our data show that the cultivated gene pool emerged from a single sub-population of wild *orientalis*. Assuming that the present genetic diversity in *orientalis* is not significantly different from the one occurring 10,000 years ago, this progenitor population was genetically structured. Indeed, two sub-populations (groups D1 and D2) were revealed by STRUCTURE (Supplementary Figure 10), and group D2 was genetically closer to all the cultivated lentil groups (Supplementary Table 5). Also, its distribution matches the earliest cultivated lentil remains in the region; however, it is a very broad distribution ranging from southern Turkey to Jordan.

Did one or several domestication events occurred in different places where wild *orientalis* sub-population D2 was found? The sympatric occurrence in the “core area” of SWA of two main ancestral populations of cultivated lentil (groups A and B) can be seen as an argument against the hypothesis of a single domestication event. The geographic distribution of our group A and group B accessions broadly correspond to the clusters K6 and K2 identified by Alo et al. (2011), respectively. Group A is mostly distributed in southeastern Turkey and Iran/Iraq, while group B is mostly present in the southern Levant. It is interesting that the four *orientalis* accessions shown in the STRUCTURE model for *K* = 4 (with *L. culinaris* accessions only) have a high proportional membership to *culinaris* group B (Supplementary Figure 7) originating from the southern Levant. This could be due to (1) these accessions becoming naturalized after escaping cultivation (as is likely the case of PI 136658), (2) introgression from cultivated lentil, or (3) ILS in a scenario where group B *culinaris* accessions descended from these particular *orientalis* accessions. Introgression from or into wild species related to cultivated crops is a documented phenomenon in sunflower (Baute et al., 2015; Hübner et al., 2019), date-palm (Flowers et al., 2019), beet (Ellstrand et al., 2013), and cassava (Bredeson et al., 2016). In Old World legumes, this has not been so thoroughly investigated. In a study of 103 accessions of sympatric wild and cultivated chickpea in the Near East, van Oss et al. (2015) found only one hybrid accession between wild and cultivated forms. Field studies indicate that the degree of outcrossing in lentil can range from 0.06 to 5.12% between cultivars and can be as high as 22% within the same cultivar, depending on environmental and genetic conditions (Horneburg, 2006). Our data indicate that gene flow is residual but was once more intense between group A and *orientalis* than between group B and *orientalis* (Figure 4). In this case, the possibility that shared alleles between southern Levant *culinaris* accessions and *orientalis* from the region results from common ancestry is reinforced. In this scenario, although group A and group B *culinaris* emerged from group D2 *orientalis*, they would have been derived from slightly different wild gene pools localized in the northern and southern Levant, respectively. Alternatively, a single domestication event could have occurred, but it would have been quickly followed by an incipient process of local adaptation to these two regions that would have resulted in these different groups. Further studies with more *orientalis* accessions will have to be carried out to elucidate the relationship between wild and cultivated plants in different regions.

*Culinaris* accessions belonging to distinct genetic clusters co-occur in almost every region and many have proportional membership to more than one cluster. This pattern was consistently observed in previous studies (Lombardi et al., 2014; Khazaei et al., 2016; Pavan et al., 2019). This mixed ancestry for some cultivated lentils can be explained by introgression throughout their cultivation history or by relaxed selection on a genetically diverse source population. In the first case, cultivation side by side of varieties descending from different source populations would have created opportunities for introgression. Pollen exchange, despite the mostly self-pollinating habit of lentils, could have resulted in hybrids. This phenomenon has been extensively studied in the case of introgression between domesticated and wild plants (Iriondo et al., 2018). However, the very low level of gene flow between the three *culinaris* groups (Figure 4D) does not lend support to this hypothesis. Rather, it is likely that, instead, lentils introduced to areas outside SWA were extracted from genetically diverse source populations. In some areas, this diversity was better preserved, whereas in others, selection, founder effects or genetic bottlenecks could have resulted in more genetically uniform landraces. A relatively fast spread of a genetically admixed source population, with no time for regional differences to emerge, explains the low isolation-by-distance (IBD) observed for all groups (Figure 5). Lentil was probably never cultivated as extensively as cereals, remaining a smaller-scale crop. As such, the selective pressure for specific traits would never have been as strong as it was for cereals. The existence of *microsperma* and *macrosperma* varieties shows that selective pressure for a basic trait, such as large seeds, was absent throughout most of the distribution range of lentils. This complementary, rather than primary, role of lentil in farming systems could explain the maintenance of some diverse populations. In other areas, pressure could have been stronger. Group C is distributed outside the Mediterranean environments where lentil emerged and has the lowest genetic diversity, which is likely to reflect a bottleneck resulting from the need of early farmers to select sturdier plants that could cope with wetter/colder climate.

We propose a model for lentil domestication where late hunter-gatherers of the PPNA were intensifying the collection of *orientalis* and experimenting with cultivation in “gardens” near their semi- or fully sedentary villages. The exploitation of wild lentil was probably more intense in southern Turkey/Syria and the Israel/Jordan areas, than in southeastern Turkey or Iraq/Iran. Archaeobotanical data show that the southern part of SWA had an early emphasis on lentils, while in the north, early farmers focused more on peas (Fuller et al., 2011). Lentil remains are only found at later stages of the Neolithic in southeastern Turkey (Girikihaciyan, 8200–7350 cal BP), Iran (Tepe Sabz, 8350–7750 cal BP), and Iraq (Jarmo, 9450–9300 cal BP), and, from the beginning of its cultivation, the diameter of the seeds is much bigger than wild *orientalis* (Zohary et al., 2012). Hence, it is plausible that the southern part of SWA was the core area of lentil domestication, with the species being introduced in an advanced stage of domestication in other parts of SWA. Intensive exploitation of wild lentil could have been done both for human consumption and as fodder for animals such as goats, cattle, or sheep, which were themselves in the process of being domesticated (Spengler and Mueller, 2019). The high protein content of lentil would have made it particularly attractive for the latter purpose. This cultivation of wild *orientalis* in designated spaces away from other wild stands would have led to reproductive isolation that eventually would have culminated in the domesticated *culinaris.*

Slightly different wild stands in different regions (i.e., Turkey/Syria and Israel/Jordan) could have led to the emergence of groups A and B in cultivated lentil. This is likely to have been a protracted process, as evidenced by the slow change in the form and size of lentils in the archaeobotanical record (Lucas and Fuller, 2014). An exchange of wild, semi-domesticated or domesticated varieties between different human groups in the region may explain the strong admixture observed in cultivated lentils. Another possibility is that group A represents the original domesticated lentil, as shown by the low genetic distance and *F*_*ST*_ between it and group D2 *orientalis* (Supplementary Table 5). Semi-domesticated or domesticated lentils in the southern Levant would then have resulted in a regional group (group B).

**FIGURE 6:**
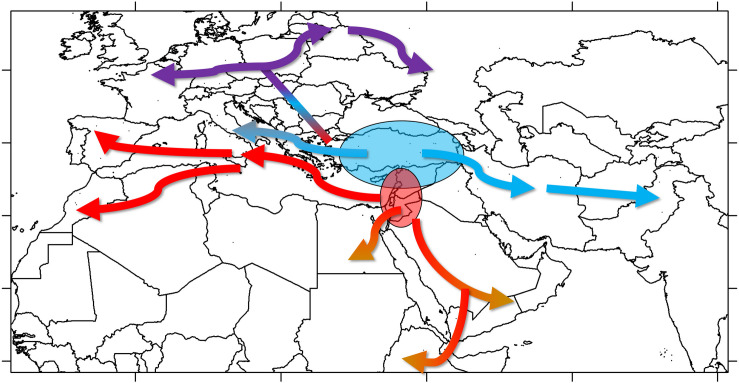
Model proposed for the spread of lentil cultivation based on genomic data.

Once established, cultivated lentil spread out of SWA. The geographic distribution of groups A and B suggests that both groups were part of the westward introduction of lentils into Europe (Figure 6). It is not clear if this happened during the Neolithic or whether it followed subsequent establishment of human populations (e.g., during the Roman or Islamic periods) bringing along these lentil varieties. Given the placement of groups B2 and C in the same clade in the phylogenetic tree, it is clear they share a most recent common ancestor, meaning that group C would have emerged from group B as lentils were introduced into Central Europe. Group C would probably have resulted from selection for varieties better adapted to the colder and more humid environment that characterizes Central Europe and could have been introduced by the early farmers associated with the LBK Neolithic culture. The spread of lentils eastwards of SWA was probably different. Group A lentils, probably emerging in the northern Levant, would have been introduced to the Iraq/Iran region and from there to Central Asia and the Indian subcontinent. Group B2 lentils would have been brought into the Arabian Peninsula, and, eventually, the Horn of Africa; their appearance in the Western Mediterranean could be a late phenomenon, as suggested by the geographical distribution of groups B1 and B2 (Supplementary Figure 9). Most probably, the spread of lentil was relatively fast with low selective pressure exerted by farmers over its cultivation history, which would explain the lack of discrete regional populations and low IBD. Introductions of varieties from distant regions during historical times are likely to have occurred.

### Genetic Basis of Lentil Domestication and Spread

The GWAS based on GBS-generated SNP data has previously been used to identify loci or genes underlying domestication and adaptation, e.g., in common bean (Oladzad et al., 2019; Rau et al., 2019) and barley (Würschum et al., 2018). Our GWAS analysis identified only eight markers associated with lentil domestication or adaptation traits. This low number may be due to a small divergence between wild and domesticated accessions, a small number of genes underlying domestication syndrome traits and others involved in physiological adaption, a small number of SNPs corresponding to the genomic regions where such genes are in, or the low number of accessions used. Nevertheless, in a set of 422,101 transcriptome-based SNPs obtained from 263 accessions, Dissanayake et al. (2020) could only identify eight candidate genes associated with variable genomic regions in lentil.

In the case of domestication, we found three associated SNPs but could not identify a putative gene associated with any of them other than an uncharacterized protein expressed in chickpea (Supplementary Table 6). Regarding adaptation, “precipitation of the wettest quarter” and “maximum temperature of the warmest month” were the only environmental variables, from the four we assessed, that seemed to have left a genomic imprint. We identified associations with an amino acid permease, an ABC transporter, and a zinc finger BED domain-containing protein DAYSLEEPER. Such proteins have been implicated in plant meristem growth, disease resistance, nitrogen metabolism, and detoxification processes (Knip et al., 2013; Do et al., 2018; Zhang et al., 2020). Further investigation on these lentil genes can offer a better view on how this crop copes with abiotic stress paving the way for novel breeds.

We investigated if both alleles for each of the five adaptation-related SNPs were present in *orientalis*. If so, we could hypothesize that adaptation emerged from standing variation of the wild progenitor and, if not, that novel mutations emerged in the domesticated gene pool and were positively selected for. For four SNPs, only the reference allele was identified in *orientalis*, and for one (SC00006020_87), both alleles were detected in the wild. It is noteworthy that, for adaptation-related SNPs, the alternative allele was always found only in group B accessions, which are distributed in the driest and warmest regions.

## Conclusion

Genotyping-by-sequencing is an effective approach to study the domestication and spread of lentil. We confirmed the existence of four gene pools within the *Lens* genus, already revealed in previous studies. *Orientalis* was shown to be the sole wild progenitor of cultivated lentil (*culinaris*), with insignificant contribution from other wild species to the domesticated gene pool. Three groups were identified within cultivated lentils, and these correspond broadly to geographic regions. Lentil was likely domesticated from wild stands from somewhere between southern Turkey, to the north, and Jordan, to the south, in a protracted and incremental fashion. Two regional groups of cultivated lentils emerged in SW Asia, which further spread into different regions. A third group probably resulted from lentil cultivation expanding into Central Europe. Introgression between cultivated lentil and its wild progenitor seems to have occurred at low levels. New mutations and selection from standing variation have probably resulted in local varieties becoming adapted to harsher environments in some areas, and these make a target for lentil breeding programs.

## Data Availability Statement

The datasets generated for this study can be found in online repositories. The names of the repository/repositories and accession number(s) can be found below: https://www.ebi.ac.uk/arrayexpress/, E-MTAB-9222.

## Author Contributions

HRO: conceptualization, plant germination and DNA extraction, and project supervision. HRO, ML, ID, and ATM: methodology. ML, HRO, and ID: data analysis. ML and HRO: writing—original draft. ML, ID, and ATM: writing—review and editing. All authors contributed to the article and approved the submitted version.

## Conflict of Interest

The authors declare that the research was conducted in the absence of any commercial or financial relationships that could be construed as a potential conflict of interest.

## Footnotes

1 http://www.fao.org/faostat/en/#data/QC (last accessed in June 2020).

2 www.ebi.ac.uk/arrayexpress

3 *Cicer arientinum*:https://www.ncbi.nlm.nih.gov/assembly/GCF_000331145.1/.

4 http://www.phylo.org/ (last accessed in June 2020).

5 https://worldclim.org/data/bioclim.html (last accessed in May 2020).

6 https://blast.ncbi.nlm.nih.gov/ (last accessed in May 2020).

## Supplementary Material

Supplementary Figure 1 PCA under different filtering conditions (number of SNPs included under brackets). **(A)** Raw data without filtering (152,076). **(B)** Raw data without missing data (5,525). **(C)** Minimum coverage of 5×, MAF ≥ 0.05 (87,647). **(D)** Minimum coverage of 5×, MAF ≥ 0.05 without missing data (4,421). **(E)** Minimum coverage of 8×, MAF ≥ 0.05 (57,138). **(F)** Minimum coverage of 8×, MAF ≥ 0.05 without missing data (1,426). **(G)** Minimum coverage of 8×, MAF ≥ 0.05, observed in 2/3 of the samples (3,613). **(H)** Minimum coverage of 8×, MAF ≥ 0.05, observed in 2/3 of the samples without missing data (250). **(I)** Minimum coverage of 8×, MAF ≥ 0.10, observed in 2/3 of the samples (2,070). **(J)** Minimum coverage of 8×, MAF ≥ 0.10, observed in 2/3 of the samples without missing data (135). **(K)** Minimum coverage of 5×, MAF ≥ 0.05, observed in 2/3 of the samples (8,791). **(L)** Minimum coverage of 5×, MAF ≥ 0.05, observed in 2/3 of the samples without missing data (809). **(M)** Minimum coverage of 5×, MAF ≥ 0.10, observed in 2/3 of the samples (5,617). **(N)** Minimum coverage of 5×, MAF ≥ 0.10, observed in 2/3 of the samples without missing data (462).

Supplementary Figure 2 PCA based on the 87,647 SNPs (Minimum coverage of 5×, MAF ≥ 0.05) with accessions colored according to their original germplasm bank classification. Detailed windows show accessions considered mislabeled or to represent potential hybrids.

Supplementary Figure 3 Plot of the Q-matrixes for STRUCTURE *K* = 3 **(A)** and *K* = 7 **(B)** models of the complete panel of 190 accessions of *Lens* genotyped with 87,647 SNPs. Black arrows indicate accessions IG72553 and PI612249, mentioned in the text. Calculations of Δ*K* and LnP(*K*) as computed by STRUCTURE HARVESTER are shown on the left.

Supplementary Figure 4 Maximum likelihood phylogeny of 190 accessions of *Lens* produced by RAxML using the GTR + Γ substitution model.

Supplementary Figure 5 3D Plot of the 3 first principal components of a PCA of 190 accessions of *Lens* based on 87,647 SNPs.

Supplementary Figure 6 Plot of the *Q*-matrix for STRUCTURE *K* = 4 model of the *L. culinaris* (subsp. *orientalis* and *culinaris*) accessions genotyped with 87,647 SNPs.

Supplementary Figure 7 Proportional membership of *orientalis* accessions in the STRUCTURE *K* = 4 model with *L. culinaris* accessions only represented in a geographical map. Each pie chart corresponds to an accession in the place it was collected and each slice indicates the proportional membership to each of the four groups.

Supplementary Figure 8 Proportional membership of *culinaris* accessions in the STRUCTURE *K* = 4 model with *L. culinaris* accessions only represented in a geographical map. Each pie chart corresponds to an accession in the place it was collected and each slice indicates the proportional membership to each of the four groups.

Supplementary Figure 9 Geographical distribution of sub-populations within groups A and B of *L. culinaris* identified in the STRUCTURE model *K* = 6. Spatial interpolation is based on the *Q*-matrix of proportional memberships of individual accessions to each of the sub-populations.

Supplementary Figure 10 Geographical distribution of the two sub-populations within group D of *L. culinaris* identified in the STRUCTURE model *K* = 2, in a run that included only *orientalis* accessions. Spatial interpolation is based on the *Q*-matrix of proportional memberships of individual accessions to each of the sub-populations.

Supplementary Figure 11 Plot of the Q-matrix for STRUCTURE *K* = 4 model of the *L. culinaris* (subsp. *orientalis* and *culinaris*) accessions, including only improved breeds or accessions of unknown improvement status.

Supplementary Figure 12 Results of the four-taxon ABBA-BABA test used to detect introgression between wild *orientalis* and groups of *culinaris* **(A–C)** and between *culinaris* groups **(D)**, using *L. nigricans* as an outgroup. Groups were defined in the STRUCTURE *K* = 4 model. The black arrow indicates the groups between which gene flow is detected.

Supplementary Figure 13 Phylogeny of 190 accessions of *Lens* produced using the GTR + Γ substitution model with different rooting taxa. **(A)** Inferred phylogeny rotted with *L. ervoides*. **(B)** Inferred phylogeny rotted with *L. nigricans*.

Supplementary Table 1 List of accessions considered to be mislabeled or hybrids and why.

Supplementary Table 2 Genetic diversity measures for species and selected groups within the *Lens* genus based on a filtered dataset without any missing data (4,421 SNPs).

Supplementary Table 3 *F_ST_* (below diagonal) and Nei’s genetic distance (above diagonal) between *L. culinaris* groups defined in the STRUCTURE *K* = 4 model.

Supplementary Table 4 *F_ST_* (below diagonal) and Nei’s genetic distance (above diagonal) between *culinaris* groups defined in the STRUCTURE *K* = 4 model and the two *orientalis* sub-groups defined in the STRUCTURE *K* = 2 model with *orientalis* accessions only.

Supplementary Table 5 Markers identified in a GWAS for association between SNPs and domesticated status as well as environmental variables.

Supplementary Table 6 List of accessions used in this study, their source, geographical provenance, species indicated in the passport data, species attribution based on our population structure data, GBS statistics, improvement status and group in STRUCTURE analysis (accessions labeled “Mixed” have less than 40% of proportional membership to any group).
