# Supplementary figures and images for: The History of Lentil (Lens culinaris subsp. culinaris) Domestication and Spread as Revealed by Genotyping-by-Sequencing of Wild and Landrace Accessions
Source: Front Plant Sci. 2021 Mar 25;12:628439. doi: 10.3389/fpls.2021.628439 (PMC8030269; doi:10.3389/fpls.2021.628439)

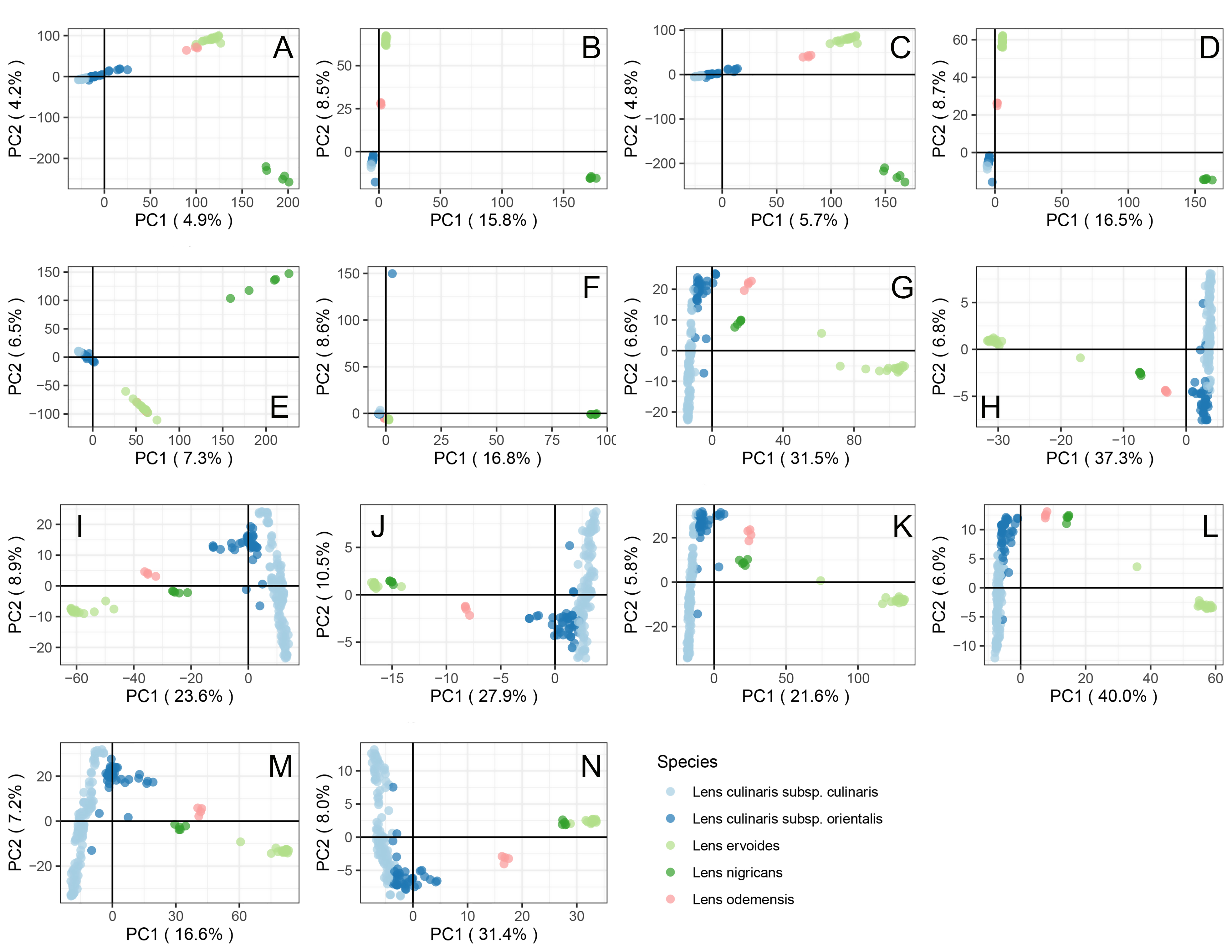

Supplement: Supplementary Figure 1 — PCA under different filtering conditions (number of SNPs included under brackets). (A) Raw data without filtering (152,076). (B) Raw data without missing data (5,525). (C) Minimum coverage of 5×, MAF ≥ 0.05 (87,647). (D) Minimum coverage of 5×, MAF ≥ 0.05 without missing data (4,421). (E) Minimum coverage of 8×, MAF ≥ 0.05 (57,138). (F) Minimum coverage of 8×, MAF ≥ 0.05 without missing data (1,426). (G) Minimum coverage of 8×, MAF ≥ 0.05, observed in 2/3 of the samples (3,613). (H) Minimum coverage of 8×, MAF ≥ 0.05, observed in 2/3 of the samples without missing data (250). (I) Minimum coverage of 8×, MAF ≥ 0.10, observed in 2/3 of the samples (2,070). (J) Minimum coverage of 8×, MAF ≥ 0.10, observed in 2/3 of the samples without missing data (135). (K) Minimum coverage of 5×, MAF ≥ 0.05, observed in 2/3 of the samples (8,791). (L) Minimum coverage of 5×, MAF ≥ 0.05, observed in 2/3 of the samples without missing data (809). (M) Minimum coverage of 5×, MAF ≥ 0.10, observed in 2/3 of the samples (5,617). (N) Minimum coverage of 5×, MAF ≥ 0.10, observed in 2/3 of the samples without missing data (462). [file Image_1.JPEG]

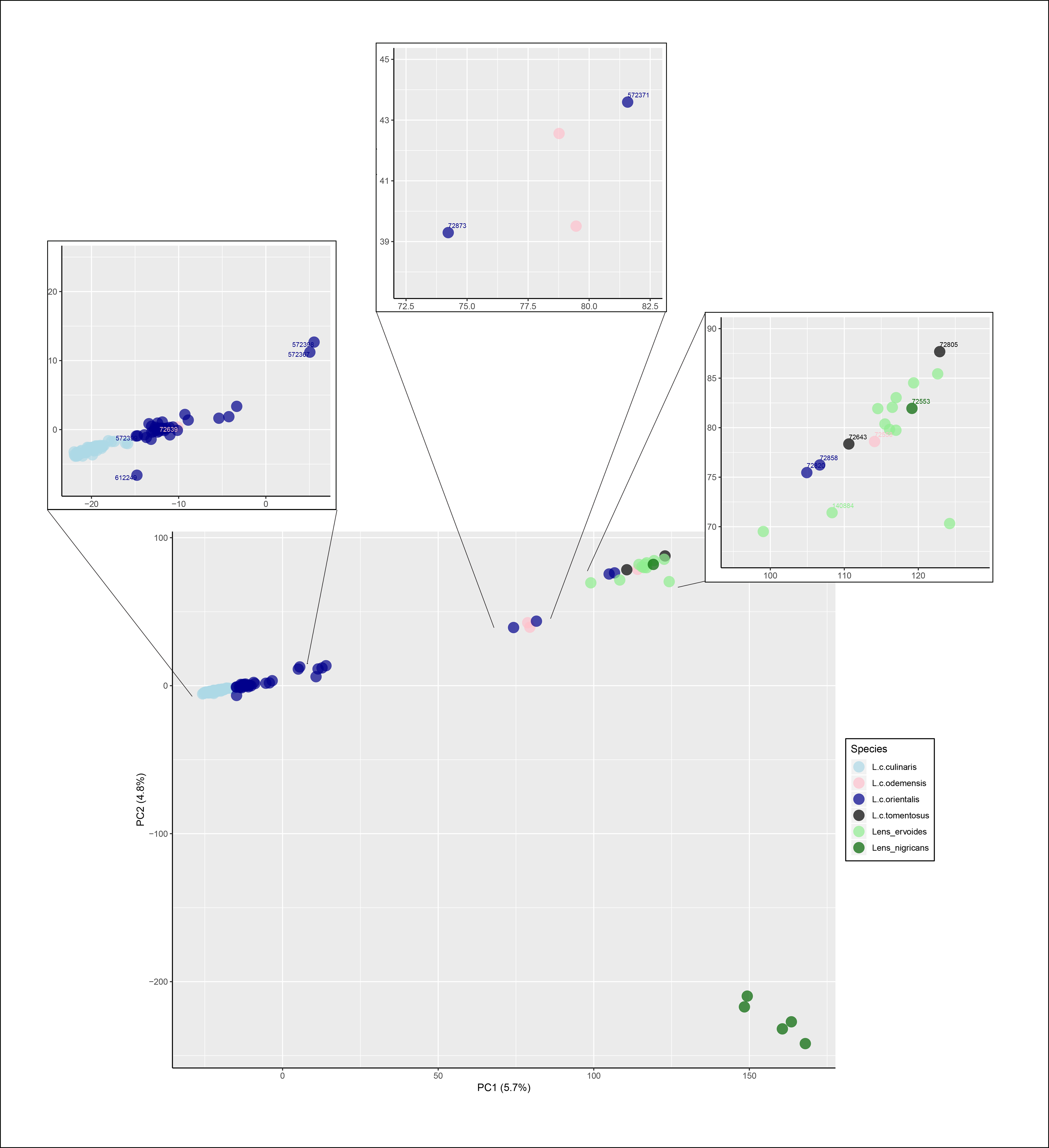

Supplement: Supplementary Figure 2 — PCA based on the 87,647 SNPs (Minimum coverage of 5×, MAF ≥ 0.05) with accessions colored according to their original germplasm bank classification. Detailed windows show accessions considered mislabeled or to represent potential hybrids. [file Image_2.JPEG]

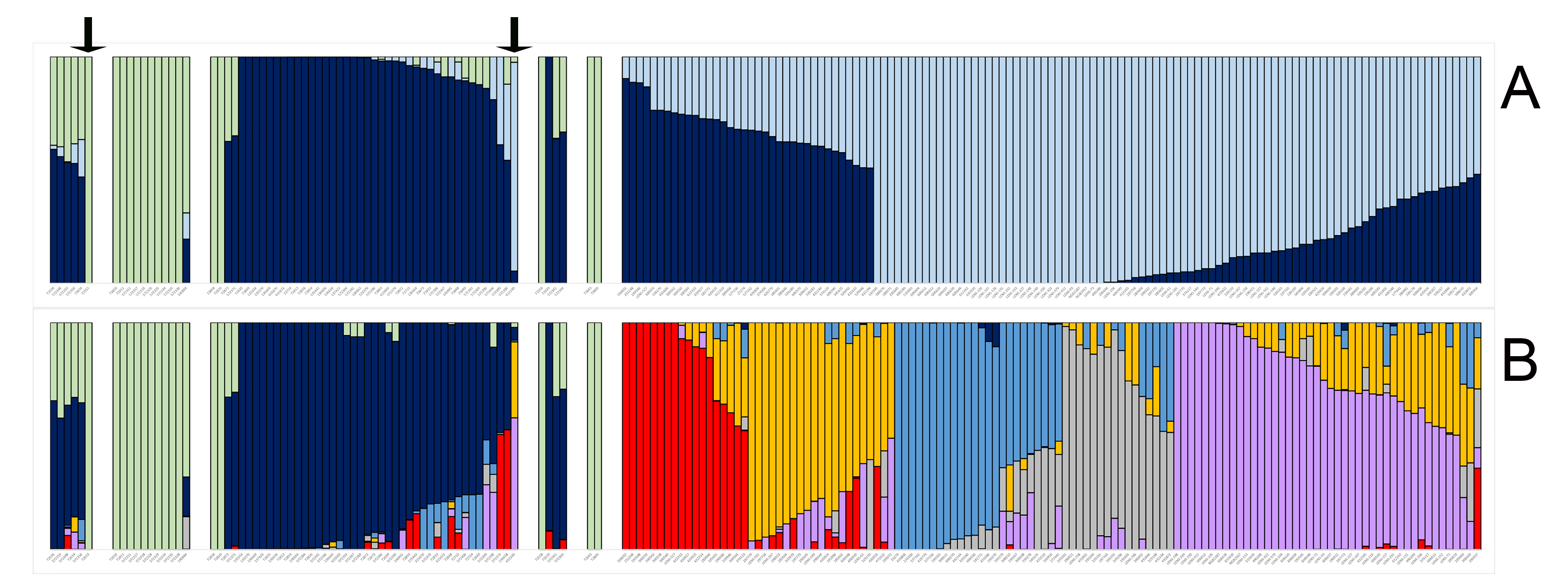

Supplement: Supplementary Figure 3 — Plot of the Q-matrixes for STRUCTURE K = 3 (A) and K = 7 (B) models of the complete panel of 190 accessions of Lens genotyped with 87,647 SNPs. Black arrows indicate accessions IG72553 and PI612249, mentioned in the text. Calculations of ΔK and LnP(K) as computed by STRUCTURE HARVESTER are shown on the left. [file Image_3.JPEG]

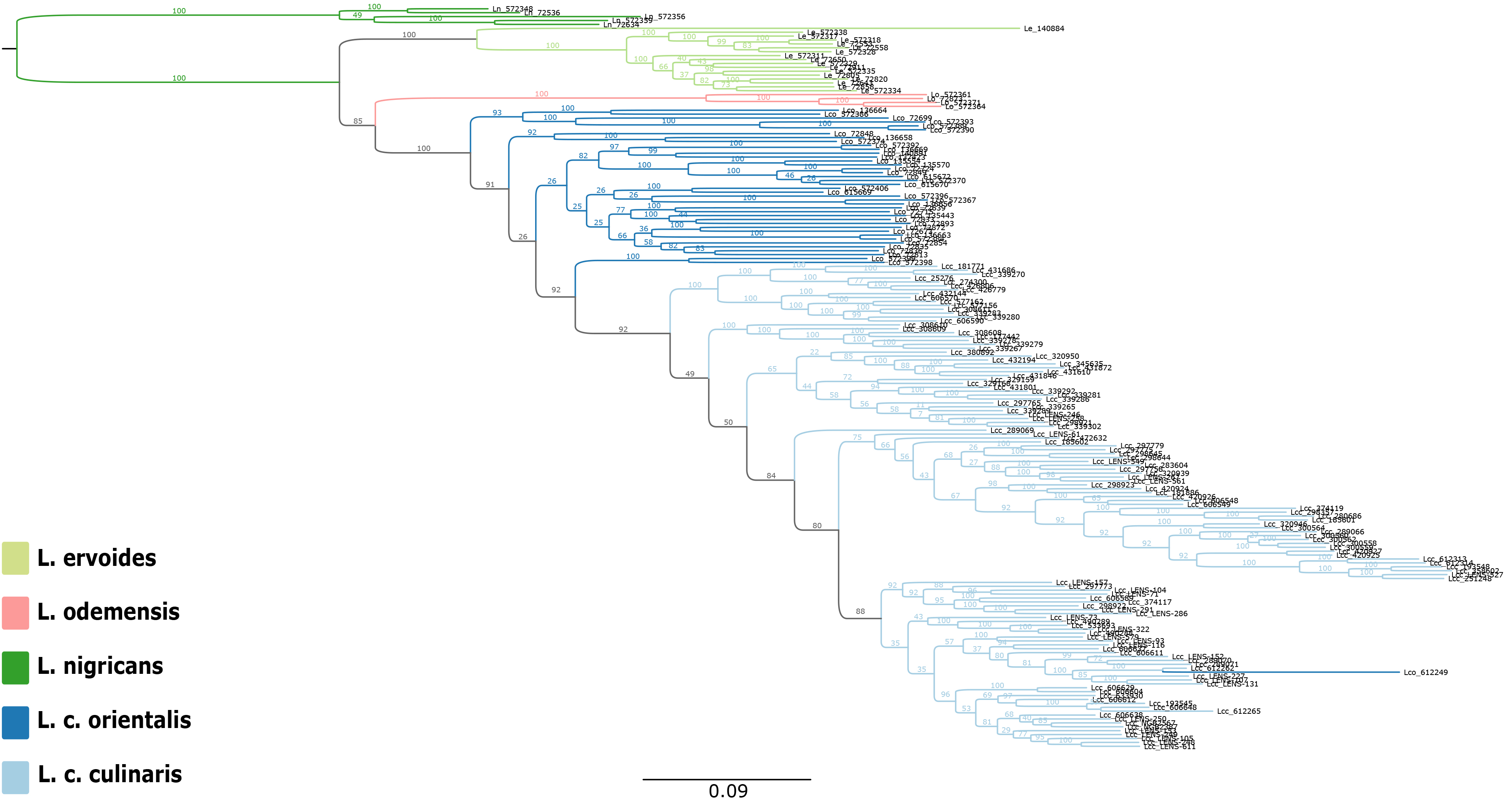

Supplement: Supplementary Figure 4 — Maximum likelihood phylogeny of 190 accessions of Lens produced by RAxML using the GTR + Γ substitution model. [file Image_4.JPEG]

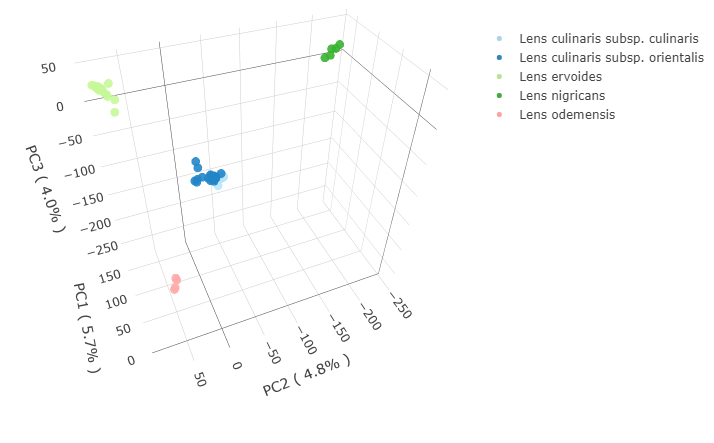

Supplement: Supplementary Figure 5 — 3D Plot of the 3 first principal components of a PCA of 190 accessions of Lens based on 87,647 SNPs. [file Image_5.JPEG]

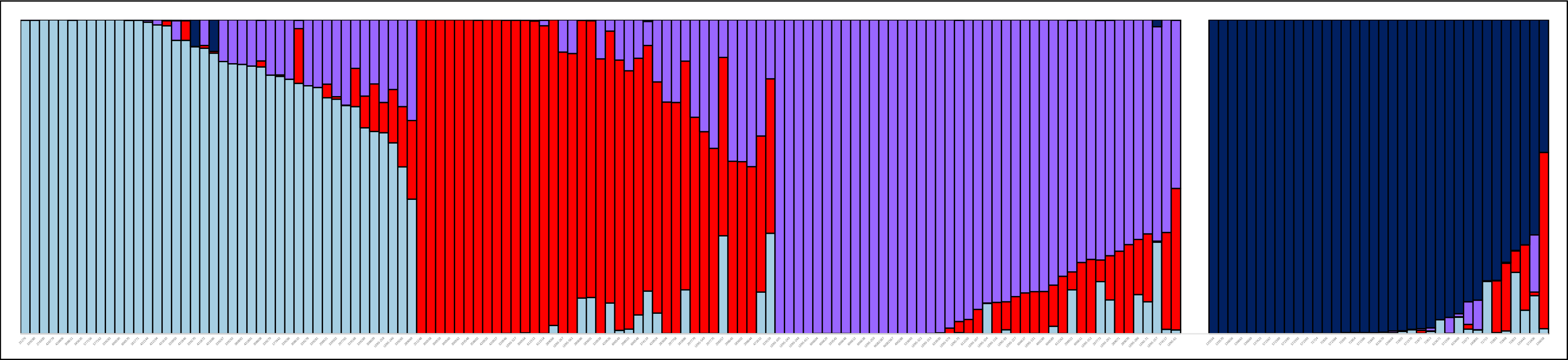

Supplement: Supplementary Figure 6 — Plot of the Q-matrix for STRUCTURE K = 4 model of the L. culinaris (subsp. orientalis and culinaris) accessions genotyped with 87,647 SNPs. [file Image_6.JPEG]

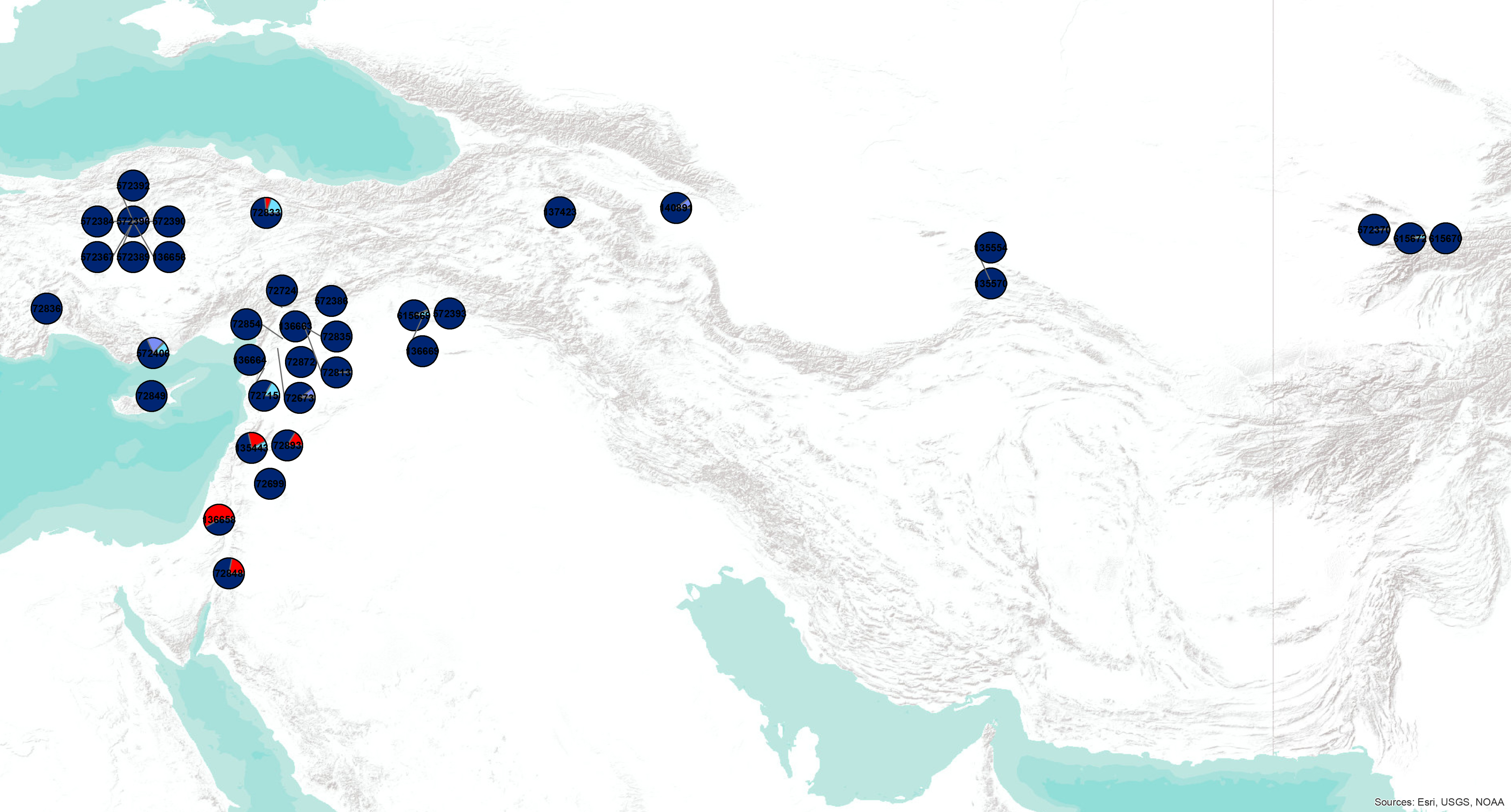

Supplement: Supplementary Figure 7 — Proportional membership of orientalis accessions in the STRUCTURE K = 4 model with L. culinaris accessions only represented in a geographical map. Each pie chart corresponds to an accession in the place it was collected and each slice indicates the proportional membership to each of the four groups. [file Image_7.JPEG]

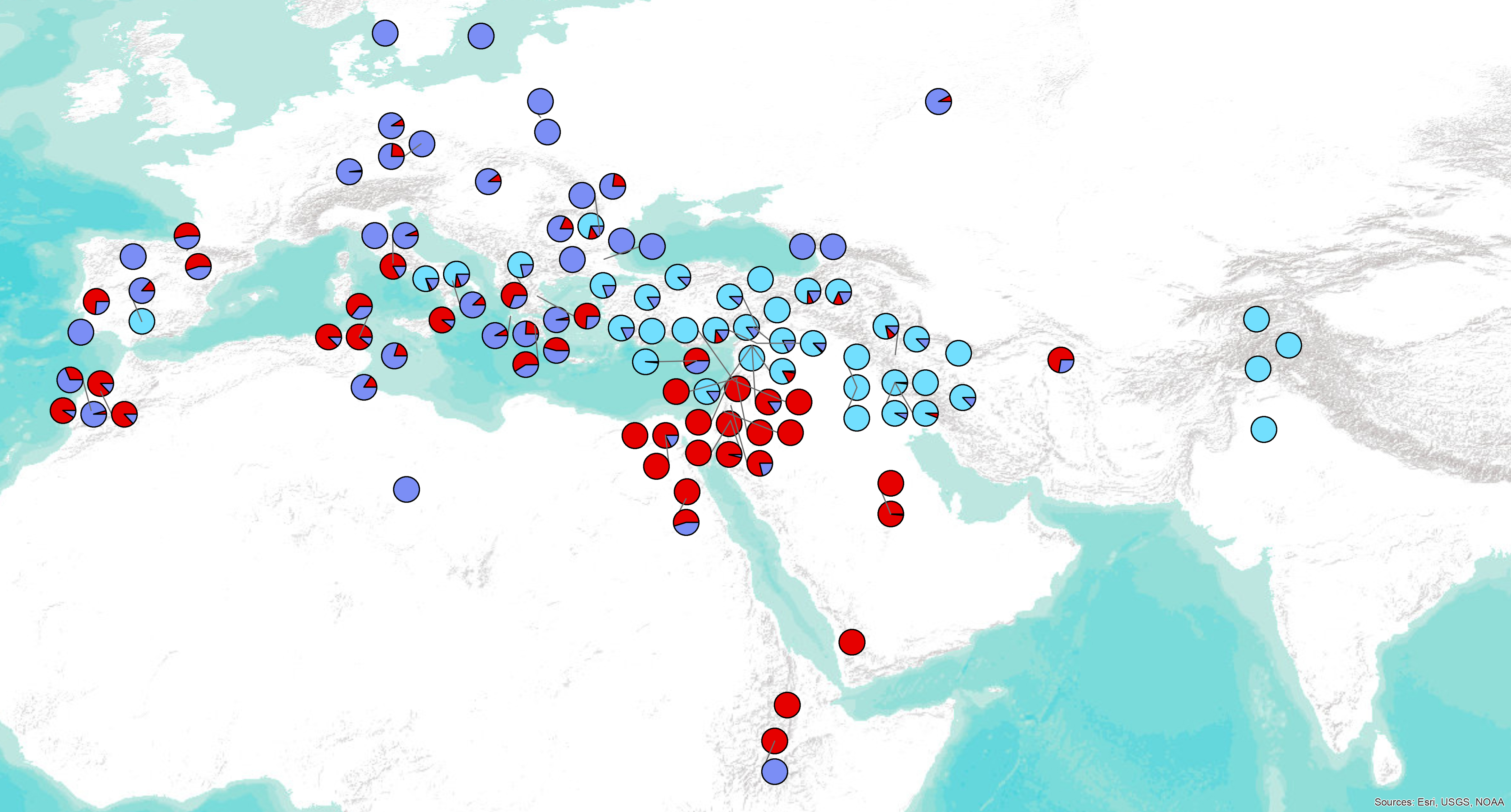

Supplement: Supplementary Figure 8 — Proportional membership of culinaris accessions in the STRUCTURE K = 4 model with L. culinaris accessions only represented in a geographical map. Each pie chart corresponds to an accession in the place it was collected and each slice indicates the proportional membership to each of the four groups. [file Image_8.JPEG]

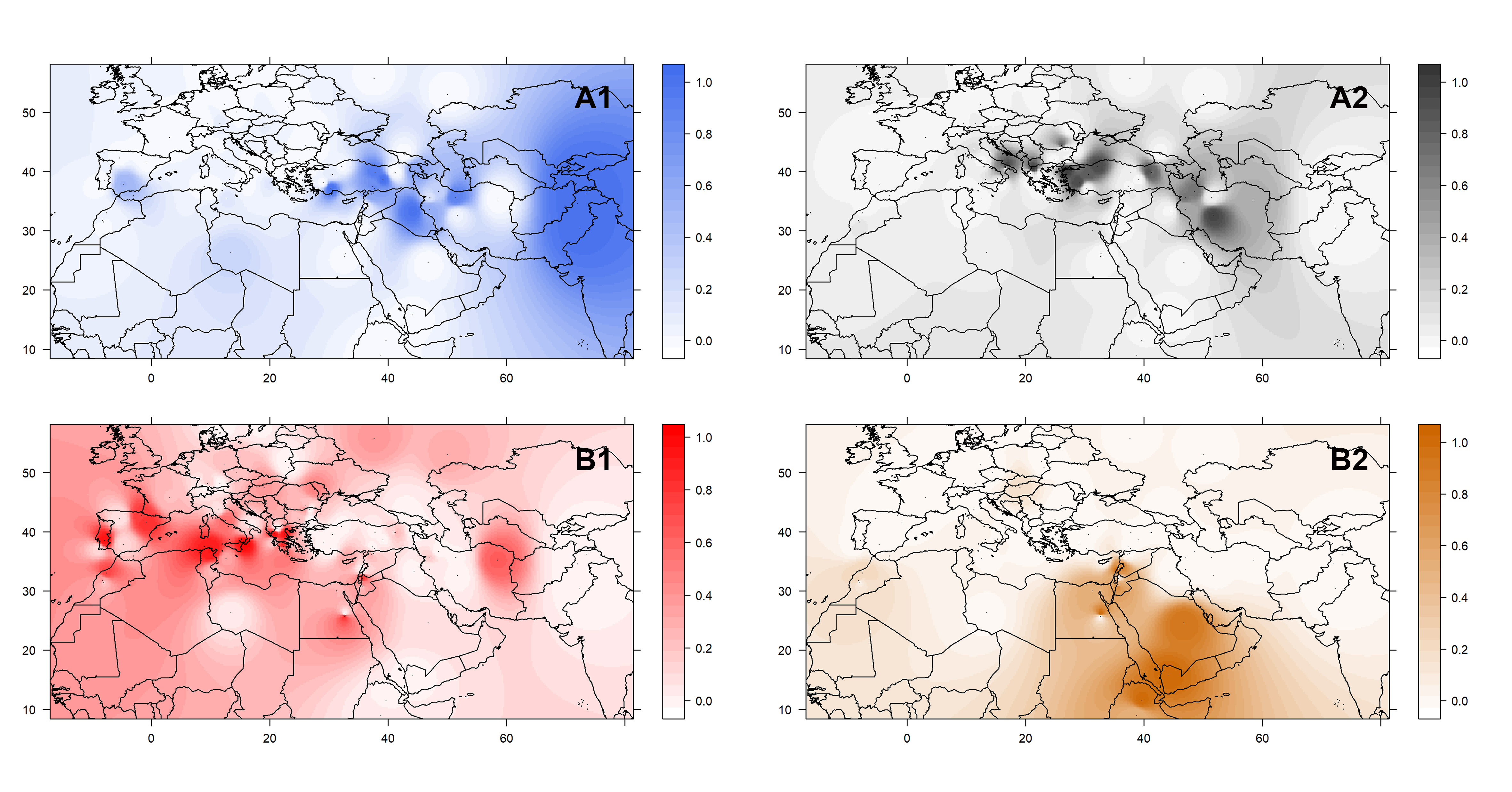

Supplement: Supplementary Figure 9 — Geographical distribution of sub-populations within groups A and B of L. culinaris identified in the STRUCTURE model K = 6. Spatial interpolation is based on the Q-matrix of proportional memberships of individual accessions to each of the sub-populations. [file Image_9.JPEG]

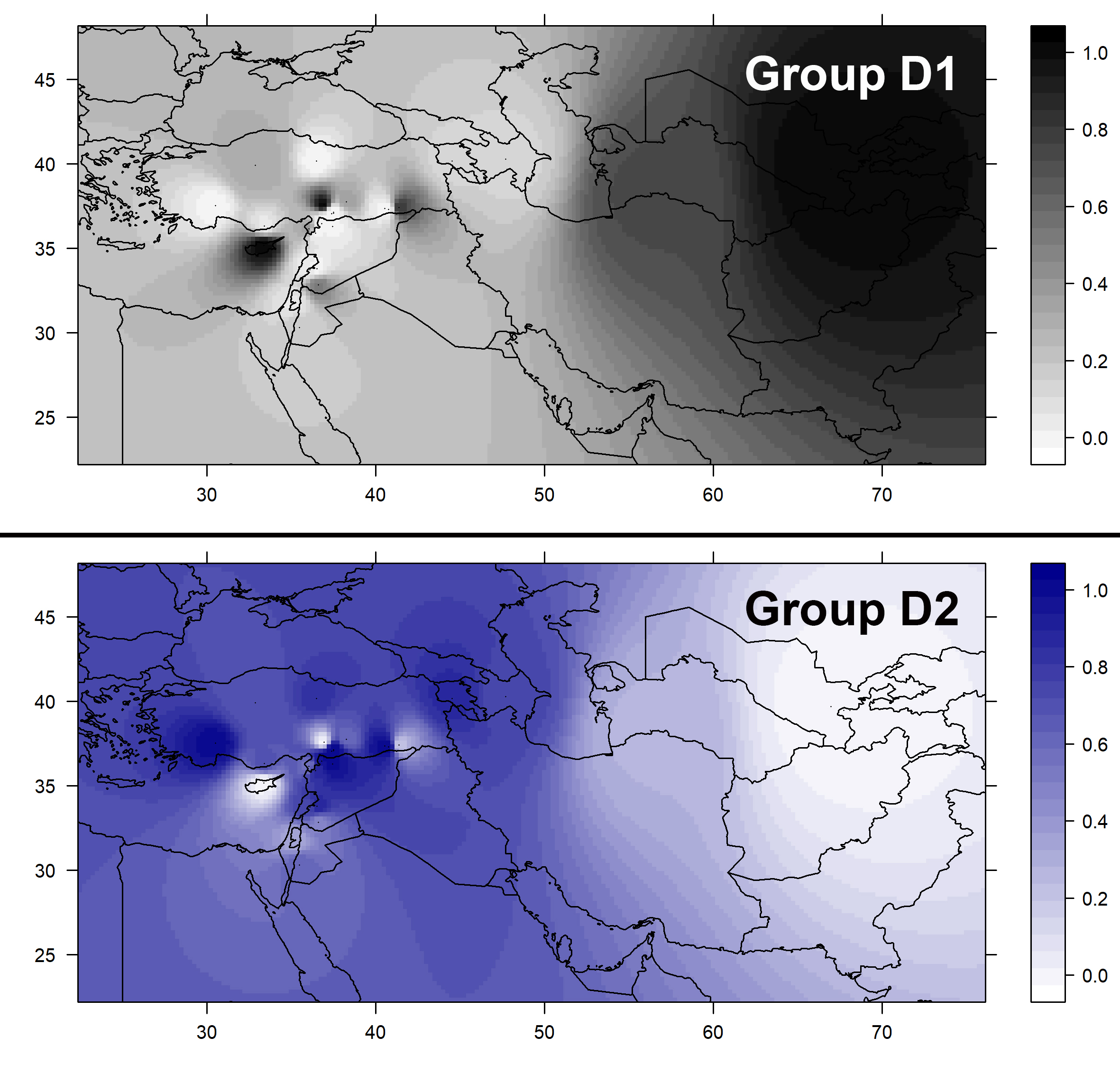

Supplement: Supplementary Figure 10 — Geographical distribution of the two sub-populations within group D of L. culinaris identified in the STRUCTURE model K = 2, in a run that included only orientalis accessions. Spatial interpolation is based on the Q-matrix of proportional memberships of individual accessions to each of the sub-populations. [file Image_10.JPEG]

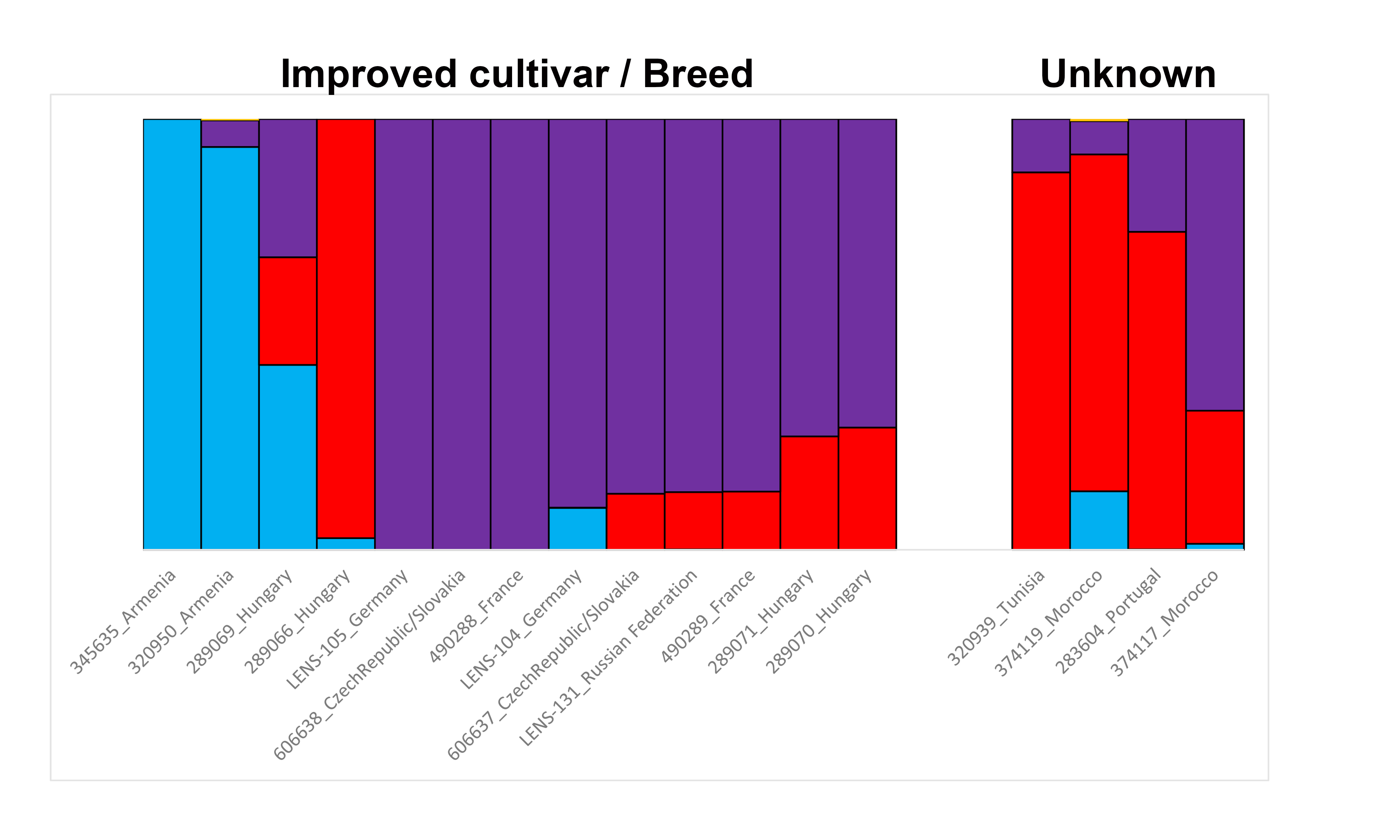

Supplement: Supplementary Figure 11 — Plot of the Q-matrix for STRUCTURE K = 4 model of the L. culinaris (subsp. orientalis and culinaris) accessions, including only improved breeds or accessions of unknown improvement status. [file Image_11.JPEG]

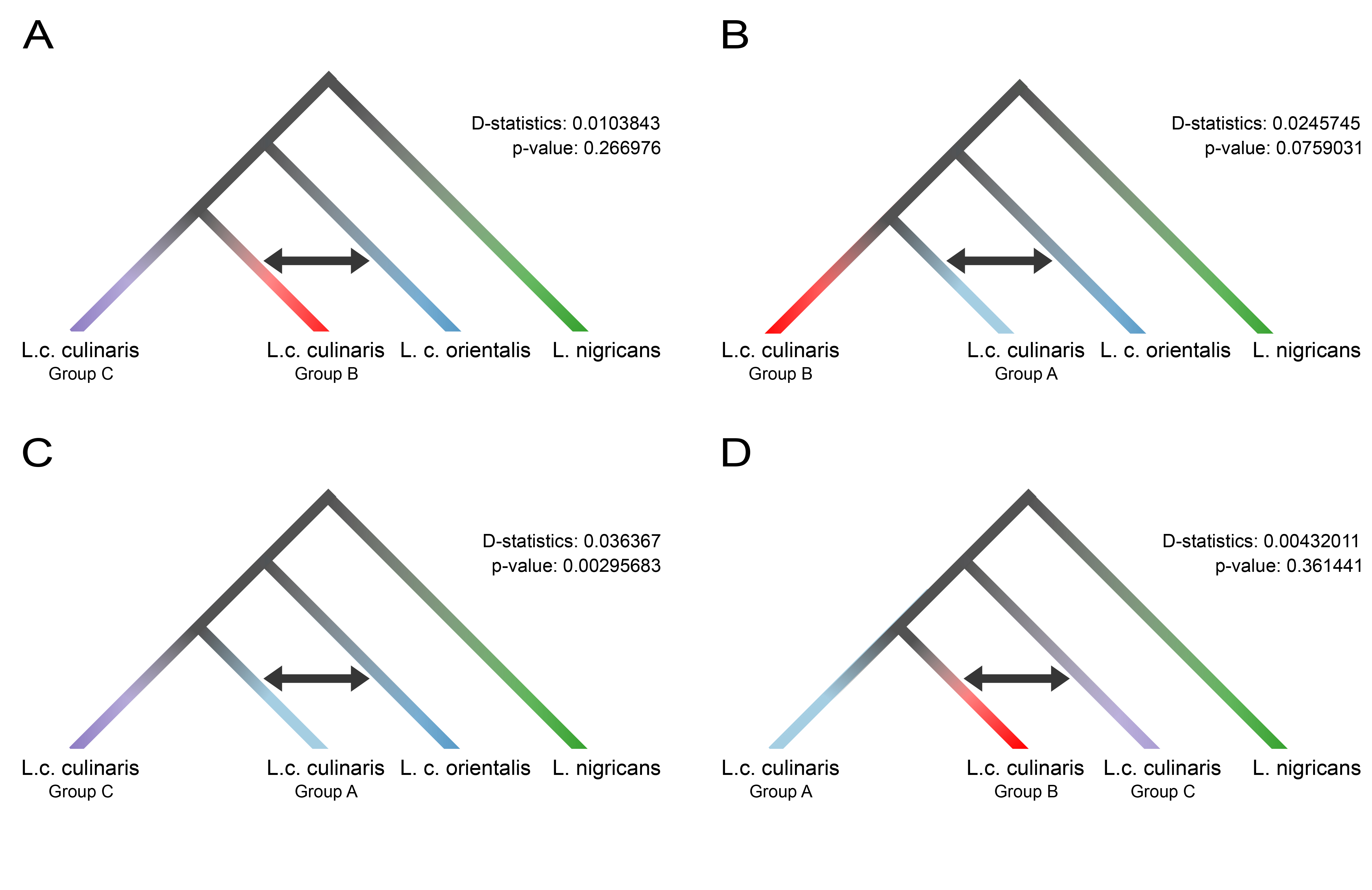

Supplement: Supplementary Figure 12 — Results of the four-taxon ABBA-BABA test used to detect introgression between wild orientalis and groups of culinaris (A–C) and between culinaris groups (D), using L. nigricans as an outgroup. Groups were defined in the STRUCTURE K = 4 model. The black arrow indicates the groups between which gene flow is detected. [file Image_12.JPEG]

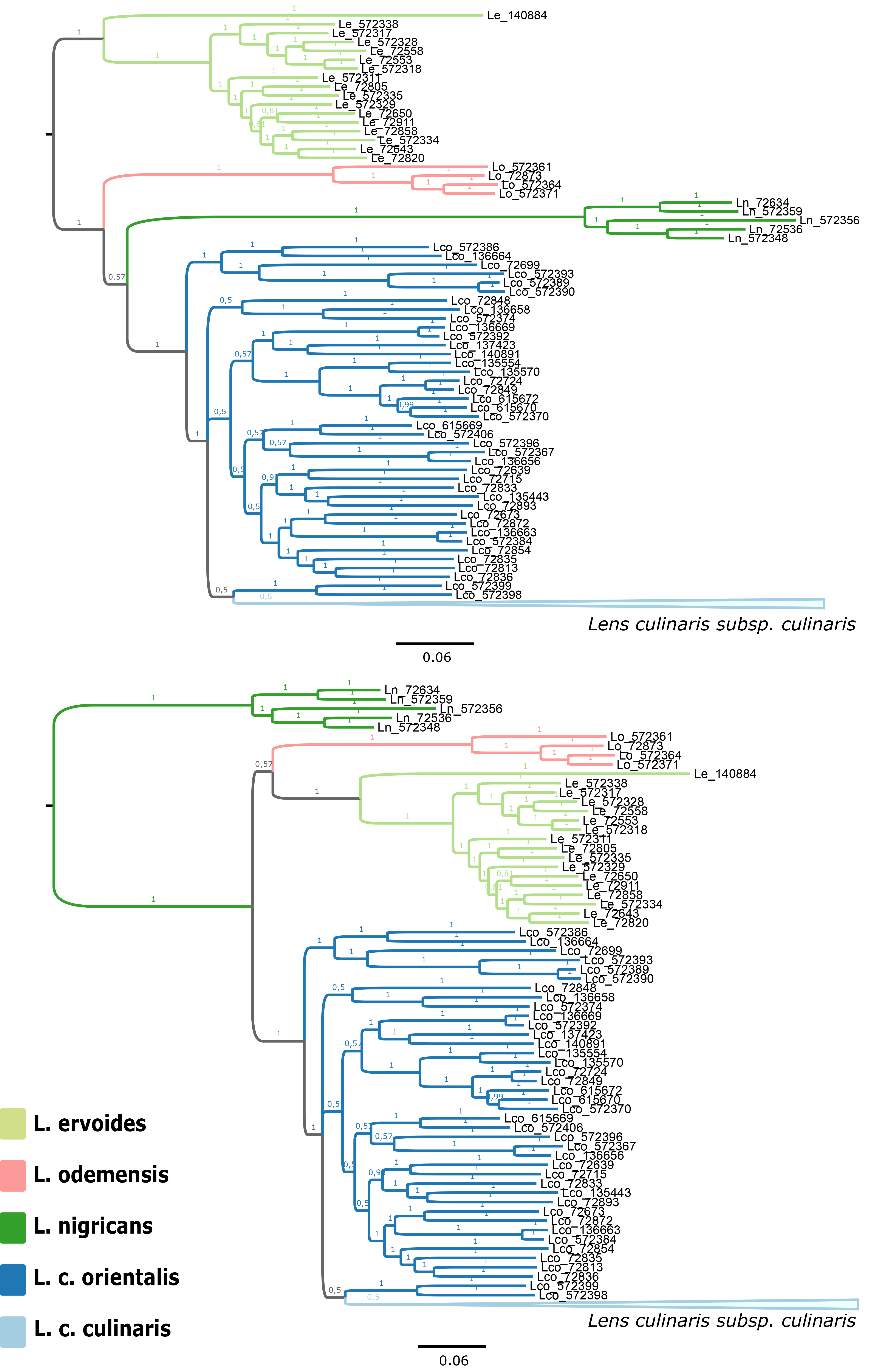

Supplement: Supplementary Figure 13 — Phylogeny of 190 accessions of Lens produced using the GTR + Γ substitution model with different rooting taxa. (A) Inferred phylogeny rotted with L. ervoides. (B) Inferred phylogeny rotted with L. nigricans. [file Image_13.JPEG]
